# Therapeutic Vaccination of Hematopoietic Cell Transplantation Recipients Improves Protective CD8 T-Cell Immunotherapy of Cytomegalovirus Infection

**DOI:** 10.3389/fimmu.2021.694588

**Published:** 2021-08-19

**Authors:** Kerstin M. Gergely, Jürgen Podlech, Sara Becker, Kirsten Freitag, Steffi Krauter, Nicole Büscher, Rafaela Holtappels, Bodo Plachter, Matthias J. Reddehase, Niels A. W. Lemmermann

**Affiliations:** Institute for Virology and Research Center for Immunotherapy (FZI) at the University Medical Center of the Johannes Gutenberg-University of Mainz, Mainz, Germany

**Keywords:** adoptive cell transfer, antiviral protection, HCMV dense bodies, subviral particles, T cell receptor transgenic cells, T cell priming, CD8^+^ T cells, vaccine

## Abstract

Reactivation of latent cytomegalovirus (CMV) endangers the therapeutic success of hematopoietic cell transplantation (HCT) in tumor patients due to cytopathogenic virus spread that leads to organ manifestations of CMV disease, to interstitial pneumonia in particular. In cases of virus variants that are refractory to standard antiviral pharmacotherapy, immunotherapy by adoptive cell transfer (ACT) of virus-specific CD8^+^ T cells is the last resort to bridge the “protection gap” between hematoablative conditioning for HCT and endogenous reconstitution of antiviral immunity. We have used the well-established mouse model of CD8^+^ T-cell immunotherapy by ACT in a setting of experimental HCT and murine CMV (mCMV) infection to pursue the concept of improving the efficacy of ACT by therapeutic vaccination (TherVac) post-HCT. TherVac aims at restimulation and expansion of limited numbers of transferred antiviral CD8^+^ T cells within the recipient. Syngeneic HCT was performed with C57BL/6 mice as donors and recipients. Recipients were infected with recombinant mCMV (mCMV-SIINFEKL) that expresses antigenic peptide SIINFEKL presented to CD8^+^ T cells by the MHC class-I molecule K^b^. ACT was performed with transgenic OT-I CD8^+^ T cells expressing a T-cell receptor specific for SIINFEKL-K^b^. Recombinant human CMV dense bodies (DB-SIINFEKL), engineered to contain SIINFEKL within tegument protein pUL83/pp65, served for vaccination. DBs were chosen as they represent non-infectious, enveloped, and thus fusion-competent subviral particles capable of activating dendritic cells and delivering antigens directly into the cytosol for processing and presentation in the MHC class-I pathway. One set of our experiments documents the power of vaccination with DBs in protecting the immunocompetent host against a challenge infection. A further set of experiments revealed a significant improvement of antiviral control in HCT recipients by combining ACT with TherVac. In both settings, the benefit from vaccination with DBs proved to be strictly epitope-specific. The capacity to protect was lost when DBs included the peptide sequence SIINFEKA lacking immunogenicity and antigenicity due to C-terminal residue point mutation L8A, which prevents efficient proteasomal peptide processing and binding to K^b^. Our preclinical research data thus provide an argument for using pre-emptive TherVac to enhance antiviral protection by ACT in HCT recipients with diagnosed CMV reactivation.

## Introduction

Human cytomegalovirus (hCMV) is the prototype member of the beta-subfamily of herpesviruses (1). Primary infection is rarely diagnosed, because it passes without overt symptoms of disease in the immunologically mature, immunocompetent host. Resolution of acute, productive infection results in maintenance of the viral genome in a non-replicative state, referred to as latent infection or, briefly, “latency”. Latency is defined by the presence of reactivation-competent viral genomes in certain cell types [for an overview, see (2)] in absence of infectious virus (3). The medical relevance of hCMV infection is based on birth defects caused by congenital infection of fetuses through diaplacental virus transmission, as well as on multiple organ disease in immunocompromised patients. Major groups at risk of lethal disease from primary infection or productive reactivation from latency are recipients of solid organ transplantation (SOT) and hematopoietic cell transplantation (HCT) [for clinical reviews, see (4–6)]. This report focuses on the further advancement of an established mouse model of experimental HCT and murine cytomegalovirus (mCMV) infection [for reviews on the model, see (7–9)] aiming at a preclinical proof-of-concept evaluation of therapeutic vaccination (TherVac) as a new option to improve immunotherapy by adoptive cell transfer (ACT) of virus-specific CD8^+^ T cells.

HCT is the therapy of choice for aggressive forms of hematopoietic malignancies that resist standard chemotherapy. Tumor cells become wiped out by hematoablative treatment that, unavoidably, co-depletes bone marrow and the immune system. Transplanted hematopoietic (stem) cells (HC) repopulate the bone marrow stroma and differentiate into all hematopoietic cell lineages, eventually reconstituting a functional immune system. Transient immunodeficiency in the period between HCT and completed reconstitution poses a “window of risk” during which latent hCMV present in transplanted donor cells or in tissues of the recipient can reactivate to productive infection that causes histopathology resulting in organ failure. Interstitial pneumonia represents the most critical organ manifestation of reactivated infection, specifically in the context of HCT. The risk of progression to CMV disease in a latently infected recipient is primarily associated with latent viral genome load in the recipient’s tissues, so that it cannot be avoided by selection of an hCMV-negative HC donor (2, 10). Close follow-up monitoring of HCT recipients for viral DNA in the blood by quantitative PCR has become clinical routine to initiate treatment with antiviral drugs upon earliest evidence of hCMV reactivation. This strategy, which is known as “pre-emptive antiviral therapy”, aims at preventing ongoing virus replication, inter- and intra-tissue spread, and organ manifestations (6, 11, 12). Although pre-emptive antiviral therapy has significantly reduced the incidence of post-HCT CMV disease, adverse side effects of antivirals (13) and drug-refractory virus variants (14–17) have made it necessary to develop the alternative strategy of immunotherapy by ACT of virus-specific CD8^+^ T cells as the last therapeutic option. ACT aims at bridging the “protection gap” between hematoablative conditioning for HCT and the completion of endogenous reconstitution of antiviral immunity (18–21).

ACT has been the validity check for the predictive value of the mouse model of CMV infection, disease, and CD8^+^ T-cell immunotherapy in the immunocompromised host, specifically also in HCT recipients under conditions of transient immunodeficiency during ongoing hematopoietic reconstitution [reviewed in (7–9)]. Prevention of a lethal CMV organ infection by ACT of virus-specific CD8^+^ T cells was originally demonstrated in the preclinical model of mice infected with mCMV after sublethal γ-irradiation (22–24), years before ACT with cell culture-propagated CD8^+^ cytolytic T-cell lines (CTLL) specific for the hCMV tegument protein pUL83/pp65 was shown to control human infection (18, 19). The combination of experimental HCT and ACT (HCT-ACT) in the mouse model revealed that ACT not only prevents lethal organ infection and histopathology but also reduces the latent viral genome load in organs and the incidence of recurrent CMV infection (25). Showing this was possible by an experimental induction of virus reactivation, an approach that can be taken only in animal models. Addressing this question by clinical investigation would require viral genome load determinations in biopsies from HCT patients who recovered from CMV reactivation, and waiting for an unpredictable, incidental secondary immunosuppression.

More recently, ACT in the mouse model of experimental HCT and CMV infection has shown that antiviral CD8^+^ T cells not only prevent viral histopathology in organs but also preclude graft failure (26) from CMV-associated inhibition of the hematopoietic repopulation of bone marrow stroma (27–29). Another valuable insight originally contributed by the mouse model is the loss of per-cell functional activity in CTLL compared to *ex vivo* isolated and directly transferred donor CD8^+^ central memory T cells (TCM) specific for the same viral epitope, the IE1 peptide YPHFMPTNL in the specific example (30, 31). Subsequent to this, high protective antiviral activity in low-dose ACT was also reported for *ex vivo* sorted hCMV epitope-specific human TCM with stemness (32–36). Yet, a direct comparison of cohorts of ACT recipients receiving CTLL or TCM of identical epitope-specificity was, of course, not feasible in a controlled clinical trial. So, again, it was up to the mouse model to have provided proof-of-concept.

The source of virus-specific CD8^+^ T cells used in clinical ACT is usually a CMV-experienced, latently infected donor, ideally, the HCT donor who is matched to the HCT recipient for avoiding a graft-*versus*-host (GvH) response against MHC (in humans, HLA) antigens. Thus, HCT-ACT donor and recipient usually share antigen-presenting MHC class-I molecules. Preferably, however, the HCT donor should be CMV-negative to avoid a contribution of transplanted latently infected hematopoietic cells to the risk of reactivation. Besides this, in clinical practice, optimized donor-recipient matching always has priority over donor CMV-status. In cases of a CMV-naïve HCT-ACT donor, CD8^+^ cells can be transduced with an engineered T-cell receptor (TCR) specific for MHC class-I-presented antigenic viral peptide to generate CMV-TCR transgenic cells for ATC (37, 38). Again, the mouse model provided proof-of-concept by showing that ACT of hCMV-TCR transgenic human CD8^+^ T cells protects HLA-transgenic mice infected with a recombinant mCMV engineered to express an antigenic peptide of hCMV (39, 40).

Although Odendahl et al. (35) reported on a clinical-scale cell isolation, the logistics for providing sufficient cell numbers, in particular of CMV-TCR transgenic CD8^+^ T cells generated from CMV-negative donors, remains demanding, and has so far precluded CMV-specific ACT from becoming clinical routine. Here, we further developed the mouse model to pursue the concept of improving the efficacy of low-dose ACT in CMV-infected HCT recipients by further expanding limited numbers of transferred donor CD8^+^ T cells within the HCT recipient by TherVac. We employed the highly versatile model of TCR-transgenic OT-I CD8^+^ T cells, which are specific for the ovalbumin (OVA)-derived antigenic peptide SIINFEKL (41, 42), for investigating the potential of TherVac to enhance the efficacy of ACT. Specifically, recipients of HCT and OT-I ACT were infected with a recombinant mCMV expressing SIINFEKL (mCMV-SIINFEKL), and TherVac was performed with recombinant hCMV dense bodies (DB-SIINFEKL) containing SIINFEKL within tegument protein pUL83/pp65. DBs were chosen for TherVac, because they represent non-infectious, enveloped and thus fusion-competent subviral particles capable of activating dendritic cells (DC) and delivering antigens into the cytosol for processing and presentation in the MHC class-I pathway (43–46). Our data show that TherVac by DB-SIINFEKL drives the proliferation of transferred OT-I cells in lymphoid tissues of ACT recipients and that equivalence in the efficacy of antiviral control in HCT recipients is reached with a significantly lower number of OT-I cells when ACT and TherVac are combined.

## Materials and Methods

### Cells and Mice

Murine embryonic fibroblasts (MEF) were prepared and cultivated in minimal essential medium (MEM, Thermo Fisher Scientific), supplemented with 10 % fetal calf serum (FCS, Thermo Fisher Scientific) and Penicillin/Streptomycin (Thermo Fisher Scientific), by standard protocol (47). Primary human foreskin fibroblasts (HFF) were grown in MEM supplemented with 10 % FCS, 2 mM L-glutamine, 50 ng gentamicin ml^−1^, and 0.5 ng basic fibroblast growth factor ml^−1^ (bFGF, Thermo Fisher Scientific).

Female C57BL/6 (8-week-old) mice were purchased from Harlan Laboratories and were housed under specified pathogen-free (SPF) conditions in the Translational Animal Research Center (TARC) of the University Medical Center of the Johannes Gutenberg-University Mainz. TCR-transgenic OT-I mice (42) were bred and housed in the TARC under SPF conditions.

### Generation of Recombinant mCMVs

Recombinant viruses mCMV-SIINFEKL and mCMV-SIINFEKA were generated by two-step replacement BAC mutagenesis in the mCMV BAC plasmid pSM3frΔm157luc (48), replacing a sequence that codes for an endogenous D^d^-presented antigenic peptide in the non-essential gene *m164* with sequences coding for peptides SIINFEKL or SIINFEKA (49, 50). The recombinant mCMVs were reconstituted in MEF and were propagated for removal of BAC sequences and for amplification (51). Infectious virions were purified according to standard procedures (47). Reconstituted and purified virus derived from the parental BAC plasmid pSM3frΔm157luc served as a control virus, for the sake of brevity herein referred to as mCMV, despite features included for a multi-purpose usage not applying to this report.

### Generation of Recombinant hCMV Dense Bodies (DBs)

Recombinant DBs HB5-pp65_SIINFEKL (briefly DB-SIINFEKL) and HB5-pp65_SIINFEKA (briefly DB-SIINFEKA) were generated by using the galK positive/negative selection procedure as described (45). In essence, the DNA sequence encoding peptides SIINFEKL or SIINFEKA was integrated into the hCMV open reading frame UL83, which is contained within BAC plasmid HB5 (52), to express fusion proteins in which SIINFEKL or SIINFEKA are integrated at amino acid position W175 of the tegument protein pUL83/pp65 (53). Unmodified hCMV DB (DB-Ø) were also HB5-derived (46). Viruses were reconstituted and stocks were prepared as described (54). DBs were purified from late-stage infected HFF by glycerol-tartrate gradient ultracentrifugation (43). In one experiment, purified ovalbumin (OVA) was used (catalog number 9006-59-1; Sigma-Aldrich Chemie, Steinheim, Germany). JPT Peptide Technologies (Berlin, Germany) synthesized OVA peptide SIINFEKL.

### Adoptive Cell Transfer (ACT)

CD8^+^ T cells were isolated from spleens of 10- to 20-week-old OT-I mice (42) by immune-magnetic cell sorting. This yields an almost pure population of Vα2Vβ5 TCR-transgenic OT-I cells specific for the peptide-MHC class-I (pMHC-I) complex SIINFEKL-K^b^. For immunotherapy by ACT, these cells were infused intravenously into total-body γ-irradiated (7 Gy) C57BL/6 mice, followed by intraplantar (left hind footpad) infection of the recipients with 10^5^ plaque-forming units (PFU) of recombinant mCMVs.

### *In Vivo* Proliferation Assay

OT-I cells were fluorescence-labeled by incubation for 4 min at 37°C at a concentration of 1 × 10^7^ cells/ml with 5 µM of 5(6)-carboxyfluorescein diacetate (CFDA; Merck Darmstadt) in phosphate-buffered saline (PBS). CFDA converts intracellularly into the fluorescent dye carboxyfluorescein diacetate succinimidyl ester (CFSE). The reaction was stopped with FCS, and the cells were washed three times with PBS [(50) and references therein]. CFSE-labeled OT-I cells (10^7^) were administered intravenously into immunocompetent C57BL/6 mice. Intraplantar infection or application of DBs was performed 24 h later. At the indicated times, OT-I cells that have homed to the spleen or the popliteal lymph node (PLN) were enriched by positive immune-magnetic sorting of CD8^+^ T cells. To assess their proliferation, loss of CFSE fluorescence with every cell division was determined by cytofluorometric analysis.

### Experimental HCT

Syngeneic HCT with 9-week-old female C57BL/6 mice as bone marrow cell (BMC) donors and recipients was performed as described in greater detail previously (29, 47). In brief, hematoablative conditioning was performed by sublethal total-body γ-irradiation with a single dose of 7 Gy. Femoral and tibial donor BMC were depleted of CD8^+^ and CD4^+^ T cells, present within bone marrow capillaries, by negative immune-magnetic cell sorting. Donor hematopoietic cells (5 × 10^6^/mouse) were infused into the tail vein of the recipients at 6 h after irradiation, followed by intraplantar infection (see above).

### T-Cell Depletion

*In vivo* depletion of CD8^+^ T cells was performed by a single intravenous injection of purified monoclonal antibody (clone: YTS169.4; 1.3 mg/mouse) directed against the CD8 molecule.

### Cytofluorometric Analyses

Single-cell suspensions were prepared from lymph nodes, spleen, and lungs, as described (47, 55). Unspecific staining was blocked with unconjugated anti-FcγRII/III antibody (anti-CD16/CD32, clone 2.4G2; BD Biosciences). Cells were stained with the following antibodies for multi-color cytofluorometric analyses: ECD-conjugated anti-CD8α (clone 53-6.7, Beckman Coulter), PE-conjugated anti-TCR Vβ5.1/5.2 (clone MR9-4, BD Biosciences), and APC-conjugated anti-TCR Vα2 (clone B20.1, BD Biosciences). Peptide/epitope-specific CD8^+^ T cells were identified with APC-conjugated MHC-I dextramer H-2Kb/SIINFEKL (Immudex, Copenhagen, Denmark). For the analyses, a “live gate” was routinely set on leukocytes in the forward scatter (FSC) *versus* sideward scatter (SSC) plot. All cytofluorometric analyses were performed with flow cytometer FC500 and CXP analysis software (Beckman Coulter).

### Quantitation of Tissue Infection and T-Cell Infiltration

Infectious virus in spleen, lungs, liver, and salivary glands was quantitated in the respective organ homogenates with high sensitivity by virus plaque assay performed under conditions of “centrifugal enhancement of infectivity” [(47, 56) and references therein]. Infected cells and tissue infiltration by CD8^+^ T cells, which include OT-I cells, were detected and quantified in liver tissue sections by two-color immunohistochemistry (2C-IHC) specific for the intranuclear viral protein IE1 (red staining) and the CD8 molecule (black staining), using monoclonal antibodies Croma101 and anti-mouse CD8 (clone 4SM15, eBioscience), respectively (47, 51). The total numbers *N* of infected IE1^+^ liver cells (mostly hepatocytes) and of liver-infiltrating or liver-localizing CD8^+^ T cells were calculated according to the formula:

$$N=n\times V/V^{*}\times \;d/(D+d)$$

*n* = number of stained cells counted in a 10-mm^2^ tissue section area (mean of five independent areas);

*V* = volume of a mouse liver embedded in paraffin (mean of 10 livers = 420 mm^3^);

V* = volume of the count disc = 10 mm^2^ × *d* = 0.02 mm^3^;

*d* = thickness of the tissue section = 0.002 mm;

*D* = maximal diameter of the counted object;

*D*-CD8^+^ T cell = 0.010 mm;

*D*-IE1^+^ hepatocyte nucleus = 0.006 mm.

From this it follows that:

$$N-\text{CD}8^{+}\text{cells}=n\times 3,500;$$

$$N-IE1^{+}\text{hepatocytes}=n\times 5,250.$$

The correction term *d*/(*D*+*d*) takes into account that an object can be cut more than once by tissue sections if *d* < *D*.

### Quantitation and Avidity Distributions of Viral Epitope-Specific CD8^+^ T Cells

An 18-h IFNγ-based enzyme-linked immunospot (ELISpot) assay was used to determine the frequency of epitope-specific CD8^+^ T cells [(30, 57) and references therein]. Briefly, graded numbers of immune-magnetically purified CD8^+^ T cells were sensitized in triplicate assay cultures by EL-4 (*H-2^b^*) cells that were exogenously loaded with epitope-representing synthetic peptides at the indicated molar loading concentrations. Custom peptide synthesis with a purity of >80% was performed by JPT Peptide Technologies (Berlin, Germany). Spots, representing single cells stimulated to secrete IFNγ, were counted automatically, based on standardized criteria using ImmunoSpot S4 Pro Analyzer (Cellular Technology Limited, Cleveland, OH, USA). Avidity distributions were derived by calculation from frequencies of CD8^+^ T cells responding to stimulation by graded target cell loading concentrations of synthetic peptides (cumulative avidity distribution), as explained in greater detail previously (55, 58).

### Determination of Viral Doubling Times

Virus growth is quantitated from log-linear regression lines [log*N*(*t*) = *at* + log*N*(0)], where *N*(*t*) is an infection parameter (ordinate), such as the number of PFU or of infected cells, measured at time *t* (abscissa) after infection, *a* is the slope of the regression line, and log *N*(*0*) is its ordinate intercept. Linear regression was calculated with GraphPad Prism 6.07 (GraphPad Software). The viral doubling time (*vDT*) is calculated according to the formula: *vDT* = log2/*a*. The upper and lower 95% confidence limit values of slope *a* (determined from the ellipsoidal parameter confidence region) define the 95% confidence intervals of *vDT* (51).

### Statistics

Frequencies (most probable numbers) of cells responding in the ELISpot assay and the corresponding 95% confidence intervals were calculated by intercept-free linear regression analysis from the linear portions of regression lines, based on spot counts from triplicate assay cultures for each of the graded cell numbers seeded (30, 57). To evaluate statistical significance of differences between two independent sets of log-transformed, log-normally distributed data, the two-sided unpaired *t*-test with Welch’s correction of unequal variances was used. In case of data sets that include data below the detection limit of the assay, which excludes log-transformation, the distribution-free Wilcoxon Mann Whitney test was applied. Differences were considered as statistically significant for *P*-values of <0.05 (^*^), <0.01 (^**^), and <0.001 (^***^). Calculations were performed with GraphPad Prism 6.07 (GraphPad Software).

## Results

### Recombinant mCMV Expressing Peptide SIINFEKL Primes CD8^+^ T Cells and Drives the Proliferation of Transferred OT-I Cells in an Epitope-Specific Manner

It was the aim of this study to develop an advanced preclinical model for enhancing the efficacy of ACT as pre-emptive immunotherapy of CMV infection in HCT patients by TherVac. To build this model, its modular components were first characterized and tested individually.

As module-1 of the mouse model, we generated recombinant viruses mCMV-SIINFEKL and mCMV-SIINFEKA expressing the antigenic model peptide SIINFEKL or its non-immunogenic analog SIINFEKA, respectively. We have previously shown that point mutation L8A of the C-terminal amino acid residue destroys immunogenicity by reducing proteasomal cleavage as well as binding to the presenting MHC class-I molecule K^b^ (49). With this strategy, one can generate an optimal control for epitope-specificity, as all other infection parameters remain conserved [for a review, see (59)]. To avoid interference with viral replicative fitness, SIINFEKL/A peptide-coding sequences replaced an endogenous antigenic sequence in a non-essential viral gene. Despite this rationale of virus design, independently generated recombinant viruses are never identical in terms of replicative fitness and should be tested before use in experiments for avoiding a misinterpretation of quantitative differences. To focus on non-immunological parameters, replicative fitness for all viruses is determined by growth kinetics and the associated viral doubling time (*vDT*) in various organs of immunocompromised mice (60–62). In the specific case here, *vDT* values differed between different organs, as it was expected from previous experience, but, within each organ, control virus mCMV as well as recombinant viruses mCMV-SIINFEKL and mCMV-SIINFEKA replicated almost equivalently, as indicated by overlapping 95% confidence intervals for the *vDT* values (**Supplementary Figure S1**).

**Figure 1:**
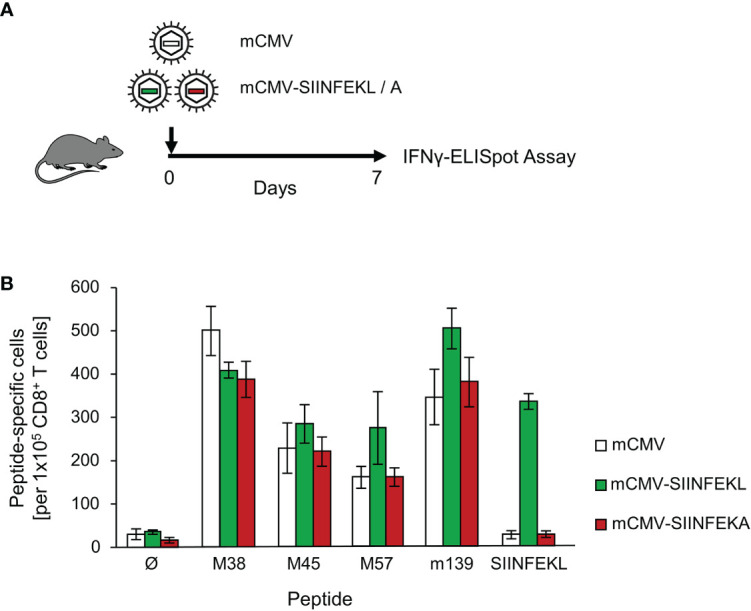
Priming of viral epitope-specific CD8^+^ T cells. **(A)** Experimental protocol. On day 7 after intraplantar infection of immunocompetent C57BL/6 mice with 10^5^ PFU of the viruses indicated, frequencies of functional, epitope-specific CD8^+^ T cells present in the spleen (from three mice pooled) were determined by an IFNγ-ELISpot assay. **(B)** Frequencies of primed cells specific for the epitopes indicated. For stimulation in the assay, EL-4 cells were exogenously loaded with the corresponding synthetic antigenic peptides at a saturating concentration of 10^-7^M. (Ø), no peptide loaded. Bars represent numbers of responding cells. Error bars indicate the 95% confidence intervals.

Based on this verified comparability in replicative fitness, we primed immunocompetent C57BL/6 (haplotype *H-2^b^*) mice by local, intraplantar infection [(57), for a discussion of priming route in vaccination, see (55)], and quantitated viral epitope-specific and functional IFNγ-secreting CD8^+^ T cells present in the spleen (**Figure 1A**, protocol; **Figure 1B**, results). As one could expect, all three viruses primed cells specific for a panel of endogenous antiviral peptides of mCMV in the *H-2^b^* haplotype (63), whereas only mCMV-SIINFEKL successfully primed SIINFEKL-specific CD8^+^ T cells, thereby verifying the prevention of immunogenicity by point mutation L8A in mCMV-SIINFEKA.

As module-2 of the mouse model, we introduced TCR-transgenic OT-I cells for performing ACT. OT-I cells express a Vα2Vβ5 TCR specific for the pMHC-I complex SIINFEKL-K^b^ (41, 42). As revealed by cytofluorometric analysis, >90% of the CD8^+^ T cells isolated from spleens of OT-I mice expressed the transgenic TCR (**Supplementary Figure S2A**).

**Figure 2:**
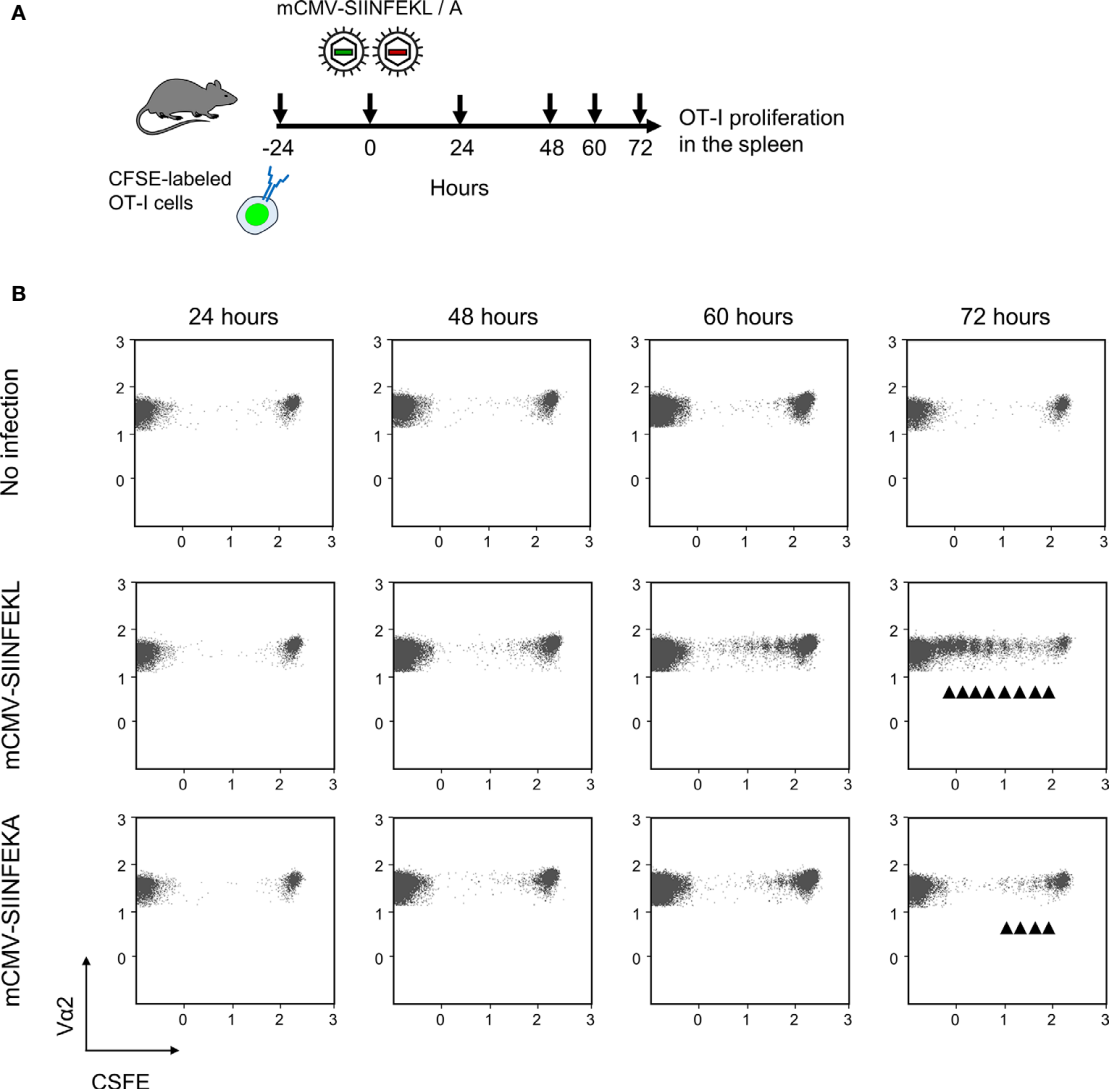
Lymphoid-tissue homing and epitope-specific proliferation of transferred OT-I cells. **(A)** Experimental protocol. CFSE fluorescence-labeled OT-I cells were transferred intravenously into immunocompetent C57BL/6 mice. Proliferation of OT-I cells recovered from the spleen (from three mice pooled) was measured at the indicated times after intraplantar infection with 10^5^ PFU of viruses mCMV-SIINFEKL or mCMV-SIINFEKA. **(B)** Cytofluorometric measurement of the loss of the fluorescence label due to the proliferation of OT-I cells over time. (Ordinate), fluorescence specific for the Vα2 TCR chain expressed by OT-I cells. (Abscissa), CFSE fluorescence. For groups of most interest, arrowheads mark cell divisions.

Combining module-1 and module-2 first in immunocompetent C57BL/6 mice, we addressed the question if an infection set on the day after intravenous ACT and at a peripheral site, specifically the mouse footpad, would drive the proliferation of CFSE fluorescence-labeled OT-I cells in lymphoid tissues in an epitope-specific manner. We have recently used this approach to document the presentation of SIINFEKL in mice latently infected with mCMV-SIINFEKL (50). In a pilot experiment, we first showed that mCMV-SIINFEKL drives the proliferation of transferred OT-I cells in the ipsilateral PLN, which is the draining regional lymph node in the case of unilateral intraplantar infection, but not in the corresponding contralateral PLN (**Supplementary Figure S3**). This shows that intravenously administered OT-I cells home to PLN and that local infection drives their proliferation. In a following experiment, we studied OT-I proliferation in the spleen in the time course and verified the epitope-specificity (for the protocol, see **Figure 2A**). Whereas proliferation was absent in the basal control group consisting of uninfected C57BL/6 mice, OT-I cells underwent several cell divisions when the recipients were infected with mCMV-SIINFEKL expressing the cognate antigenic peptide. In contrast, only few cell divisions were observed after infection with epitope mutant virus mCMV-SIINFEKA (**Figure 2B**). These findings thus indicated some epitope-independent, but infection-related proliferation of transferred OT-I cells, most likely driven by virally induced cytokines, as well as a much stronger component of an epitope-specific proliferation.

### ACT With OT-I Cells Protects Against Infection of Immunocompromised Recipients in a Cell Dose-Dependent and Epitope-Specific Manner

**Figure 3:**
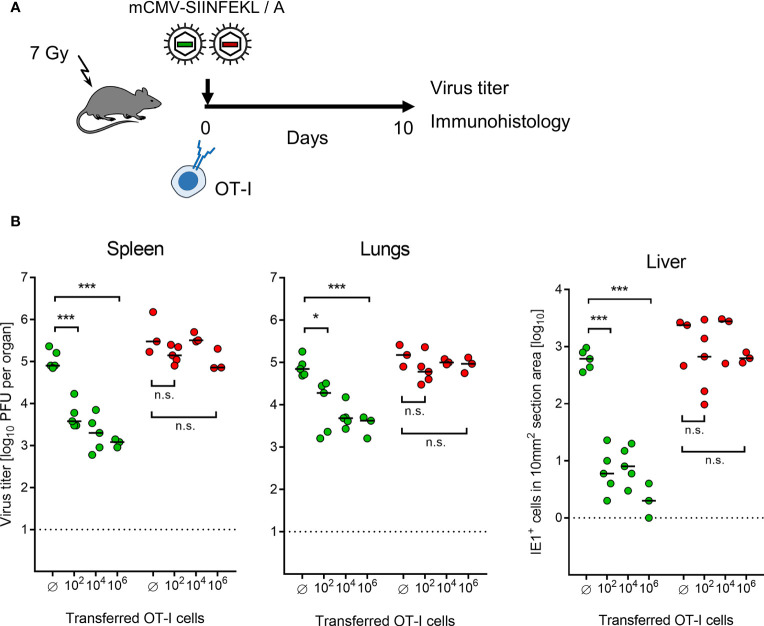
Control of organ infection by ACT of OT-I cells. **(A)** Experimental protocol. OT-I cells were transferred intravenously into immunocompromised C57BL/6 mice on the day of intraplantar infection with 10^5^ PFU of viruses mCMV-SIINFEKL or mCMV-SIINFEKA. **(B)** Virus titers in spleen and lungs, as well as numbers of infected IE1^+^ liver tissue cells in representative 10-mm^2^ section areas were determined on day 10 after infection and transfer of graded numbers of OT-I cells. (Ø) no OT-I cells transferred. Symbols (green: mCMV-SIINFEKL, red: mCMV-SIINFEKA) represent data from individually tested mice, with the median values marked. Significance levels (*) *P* < 0.05, (***) *P* < 0.001. (n.s.), not significant (*P* > 0.05).

Continuing with combining module-1 and module-2, now in ACT recipients immunocompromised by sublethal total-body γ-irradiation, the antiviral potential of pre-emptive immunotherapy by ACT with OT-I cells was tested by transferring graded numbers of OT-I cells, followed by infection with mCMV-SIINFEKL or mCMV-SIINFEKA (**Figure 3A**). Virus epitope-specific CD8^+^ T cells can only protect against infection when their functional avidity is high enough to detect pMHC-I complexes presented at the cell surface after endogenous antigen processing and presentation. In CMV infections, the demand for high avidity is tightened by the expression of immune evasion proteins that interfere with cell surface trafficking of pMHC-I complexes [for mCMV, see (49, 64, 65)]. As we have shown recently, recognition of infected cells requires a functional avidity that corresponds to the recognition of ≤10^-9^ M of exogenously loaded antigenic peptide (55). We have therefore determined the avidity distribution for OT-I cells and found that most functional cells in the OT-I population have an avidity corresponding to 10^-10^ M, and a significant proportion even to 10^-11^ M (**Supplementary Figure S2B**). Thus, OT-I cells fulfill a prerequisite for protecting against mCMV infection upon ACT despite the expression of viral immune evasion proteins in infected tissue cells of the recipients.

This prediction came true in ACT, demonstrating a cell dose-dependent protection against infection with mCMV-SIINFEKL in spleen, lungs, and liver (**Figure 3B**). Notably, low-dose ACT with just 100 OT-I cells significantly reduced the infection in all three organs tested, which is in good agreement with clinical data on low-dose ACT with streptamer-enriched hCMV-specific CD8^+^ T cells into HCT recipients, as reported by Stemberger and colleagues (33). In contrast, as a specificity control that cannot be accomplished in a clinical trial, even a 10,000-fold higher number of transferred OT-I cells failed to reduce the infection with mCMV-SIINFEKA, in which the cognate epitope is selectively missing, while all other parameters associated with infection are maintained. These include cytokine network perturbation, innate immune responses, non-cognate antigen presentation, and a general immune system remodeling.

**Figure 4:**
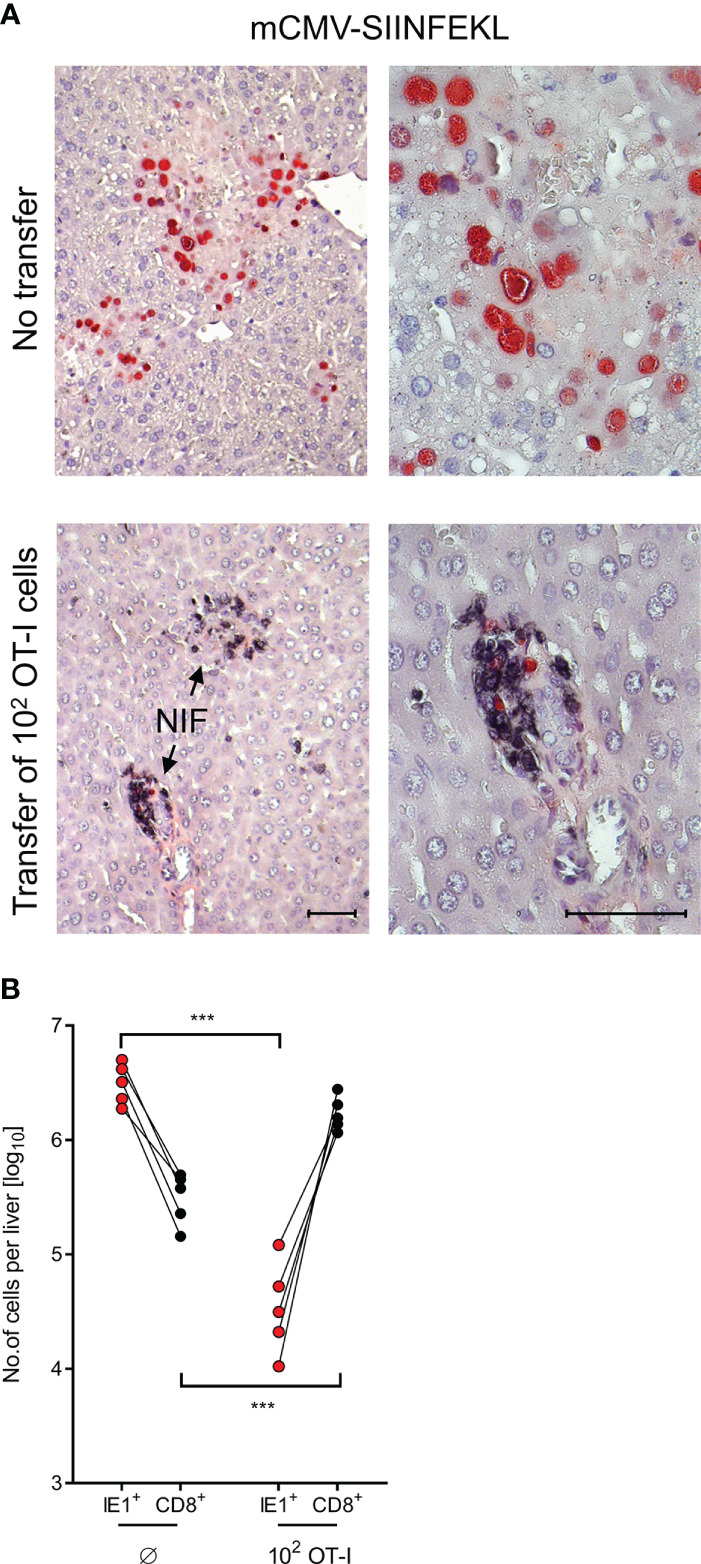
Control of liver infection by tissue infiltrating OT-I cells. **(A)** 2C-IHC images of liver tissue sections, corresponding to groups with no cell transfer and with transfer of 100 OT-I cells in Figure 3. Liver cells, mostly hepatocytes, infected with mCMV-SIINFEKL are identified by expression of the intranuclear viral protein IE1 (red staining). Residual CD8^+^ T cells and infiltrating OT-I cells are visualized by black staining of the CD8 molecule. (Left images), low-magnification overview. (Right images), resolution to greater detail by higher magnification. Bar markers, 50 μm. (NIF), nodular inflammatory foci that represent microanatomical sites where protective OT-I cells aggregate at infected liver cells to confine and resolve the infection. **(B)** Absolute numbers of infected liver cells (red symbols) and of residual CD8^+^ T cells (Ø, no transfer control group) as well as tissue-infiltrating OT-I cells (both with black symbols) were determined by quantitative 2C-IHC for five mice individually. Paired data are connected by lines. (***) *P* < 0.001.

After infection with mCMV-SIINFEKL, 2C-IHC images of liver tissue sections (**Figure 4A**) show disseminated tissue infection with extended plaque-forming clusters of infected cells, mostly of hepatocytes (61, 66), when no OT-I cells were transferred. In contrast, after transfer of as few as 100 OT-I cells, liver tissue infection was largely controlled by tissue-infiltrating OT-I cells that confine and eventually resolve the infection by aggregating around few remaining infected cells, thereby forming nodular inflammatory foci (NIF). These are micro-anatomical structures that are indicative of epitope-specific protection (31, 39, 67, 68). In the absence of the epitope, that is, after infection with mCMV-SIINFEKA, few OT-I cells are seen being randomly distributed in highly infected liver tissue, failing to form NIF (**Supplementary Figure S4**).

It appears to be obvious that protection against mCMV-SIINFEKL in different organs cannot be exerted by the 100 OT-I cells transferred, but depends on clonal expansion in the recipients (33, 34). For a minimum estimate of OT-I cell divisions, we determined the absolute numbers of OT-I cells present in the liver on day 10 after transfer of an initial number of 100 OT-I cells, and paired these numbers with the absolute numbers of infected liver cells in 5 mice analyzed by 2C-IHC individually (**Figure 4B**). A control group not receiving OT-I cells served to quantitate residual liver-resident CD8^+^ T cells for subtraction. Control of infection clearly correlated with tissue infiltration by OT-I cells. The mean number of OT-I cells per liver was 1.445 × 10^6^, which corresponds to 13–14 cell divisions. This has to be interpreted as a minimum estimate, as OT-I cells infiltrate also other tissues.

### Recombinant Dense Bodies (DBs) Prime Epitope-Specific CD8^+^ T Cells

**Figure 5:**
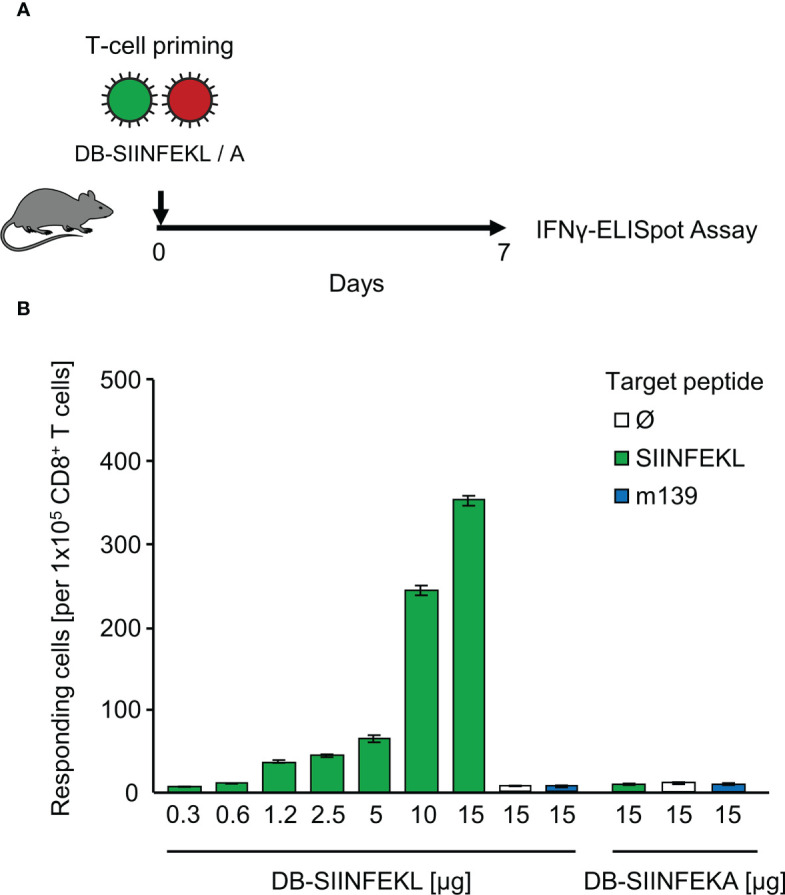
Epitope-specific priming of CD8^+^ T cells by recombinant dense bodies (DBs). **(A)** Experimental protocol. On day 7 after intraplantar application of graded μg-doses of DB-SIINFEKL or DB-SIINFEKA to immunocompetent C57BL/6 mice, frequencies of functional, viral epitope-specific CD8^+^ T cells recovered from the spleen (from three mice pooled) were determined by an IFNγ-ELISpot assay. **(B)** Frequencies of primed cells specific for the viral epitopes indicated. For stimulation in the assay, EL-4 cells were exogenously loaded with the corresponding synthetic antigenic peptides at a saturating concentration of 10^-7^ M. (Ø), no peptide loaded. Bars represent numbers of responding cells. Error bars indicate the 95% confidence intervals.

As module-3 of the mouse model, we introduced hCMV DBs for future use in TherVac. Recombinant DBs were engineered to contain peptides SIINFEKL or SIINFEKA integrated within the protein pUL83/pp65, which is a tegument protein of hCMV virions and the major component of DBs (69). The potential of DB-SIINFEKL for priming of SIINFEKL-specific CD8^+^ T cells in immunocompetent C57BL/6 mice was tested by intraplantar application of graded doses of purified DBs, and frequencies of functional, IFNγ-secreting CD8^+^ T cells were determined on day 7 in the spleen (**Figure 5A**). The response increased steadily with increasing doses of DBs and was strictly epitope-specific. Only DB-SIINFEKL, but not DB-SIINFEKA, induced SIINFEKL-K^b^ specific CD8^+^ T cells. An unrelated mCMV peptide, namely, peptide m139 that is also presented by K^b^, was not recognized after application of either type of DB (**Figure 5B**).

### Vaccination With Recombinant DBs Protects Immunocompetent Mice Against a High-Dose Challenge Infection

**Figure 6:**
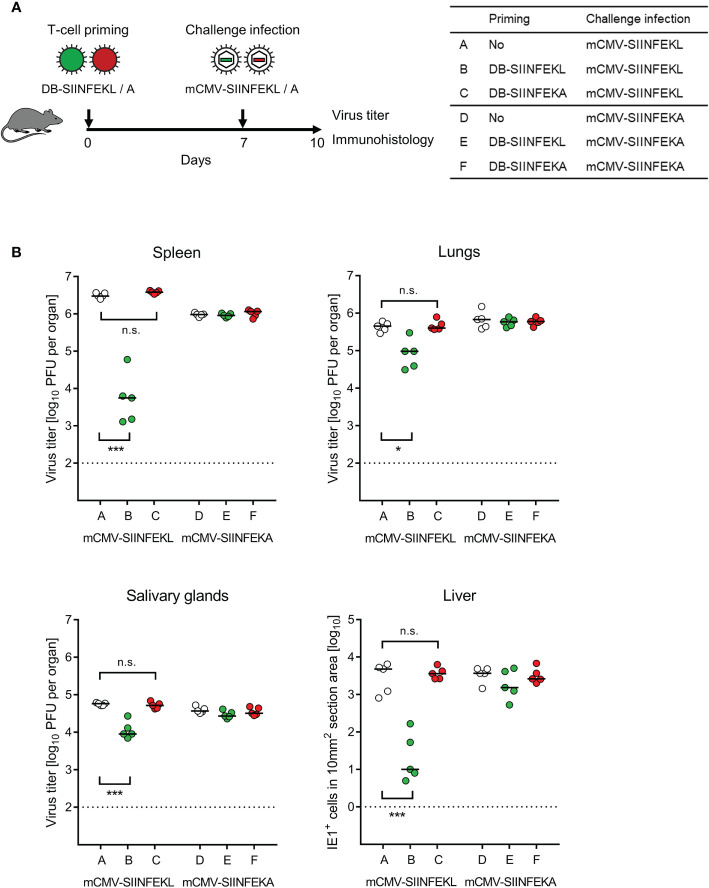
Protection against challenge infection by vaccination with recombinant DBs. **(A)** Sketch of the protocol and table of experimental groups A–F. Immunocompetent C57BL/6 mice were primed (vaccinated) by intraplantar application of 20-μg doses of DB-SIINFEKL or DB-SIINFEKA. High-dose (10^6^ PFU) intravenous challenge infections with mCMV-SIINFEKL or mCMV-SIINFEKA were performed on day 7 after priming (vaccination). Control of organ infection was assessed on day 10. **(B)** Virus titers in spleen, lungs, and salivary glands, as well as numbers of infected IE1^+^ liver tissue cells in representative 10-mm^2^ section areas. Symbols (open circles: no vaccination; green closed circles: vaccination with DB-SIINFEKL; red closed circles: vaccination with DB-SIINFEKA) represent data from individually tested mice, with the median values marked. Significance levels (*) *P* < 0.05, (***) *P* < 0.001. (n.s.), not significant (*P* > 0.05). (Dotted lines), detection limits of the assays.

Combining module-1 and module-3, we tested the capacity of DBs to serve as a prophylactic intraplantar vaccine for protection against a subsequent high-dose intravenous challenge infection of immunocompetent C57BL/6 mice (**Figure 6A**). Compared to a control group with no vaccination, intraplantar priming (recall **Figure 5**) with DB-SIINFEKL significantly reduced the replication of challenge virus mCMV-SIINFEKL in all organs tested, but not of challenge virus mCMV-SIINFEKA lacking the cognate epitope. Accordingly, vaccination with the epitope-deletion variant DB-SIINFEKA failed to protect against challenge infection with either virus (**Figure 6B**).

**Figure 7:**
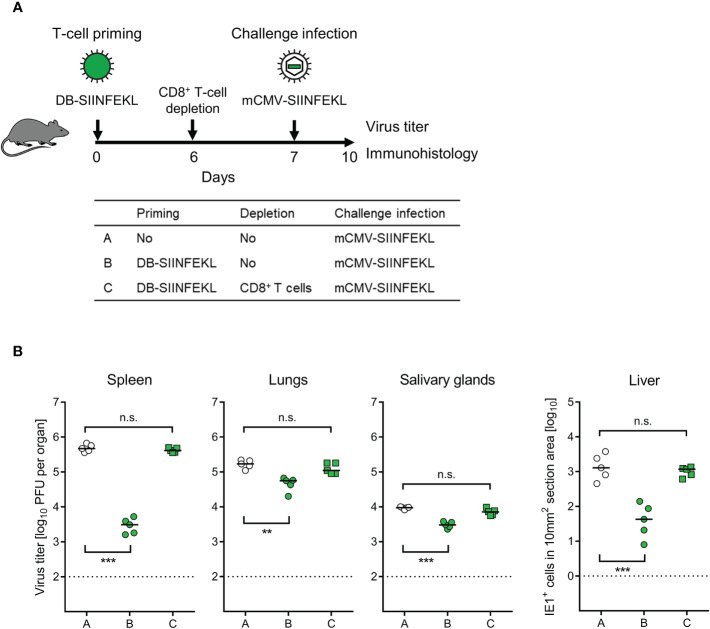
Identification of the protective T-cell subpopulation. **(A)** Sketch of the protocol and table of experimental groups A–C. Immunocompetent C57BL/6 mice were primed (vaccinated) by intraplantar application of 20-μg doses of DB-SIINFEKL. CD8^+^ T cells were depleted on day 6, followed by high-dose (10^6^ PFU) intravenous challenge infection with mCMV-SIINFEKL on day 7. Control of organ infection was assessed on day 10. **(B)** Virus titers in spleen, lungs, and salivary glands, as well as numbers of infected IE1^+^ liver tissue cells in representative 10-mm^2^ section areas. Symbols (open circles: no vaccination; green closed circles: vaccination with DB-SIINFEKL; green closed squares: vaccination with DB-SIINFEKL followed by depletion of CD8^+^ T cells) represent data from individually tested mice, with the median values marked. Significance levels (**) *P* < 0.01, (***) *P* < 0.001, (n.s.), not significant (*P* > 0.05). Dotted lines, detection limits of the assays.

To identify the protective cell type primed by DB-vaccination, we depleted CD8^+^ T cells 6 days after priming with DB-SIINFEKL and one day before a high-dose intravenous challenge infection with mCMV-SIINFEKL (**Figure 7A**). Again, confirming the results of the experiment shown above (**Figure 6**), vaccination with DB-SIINFEKL significantly reduced virus replication in all organs tested when compared to the control group with no vaccination. This protection was abolished by depletion of CD8^+^ T cells shortly before challenge infection (**Figure 7B**).

**Figure 8:**
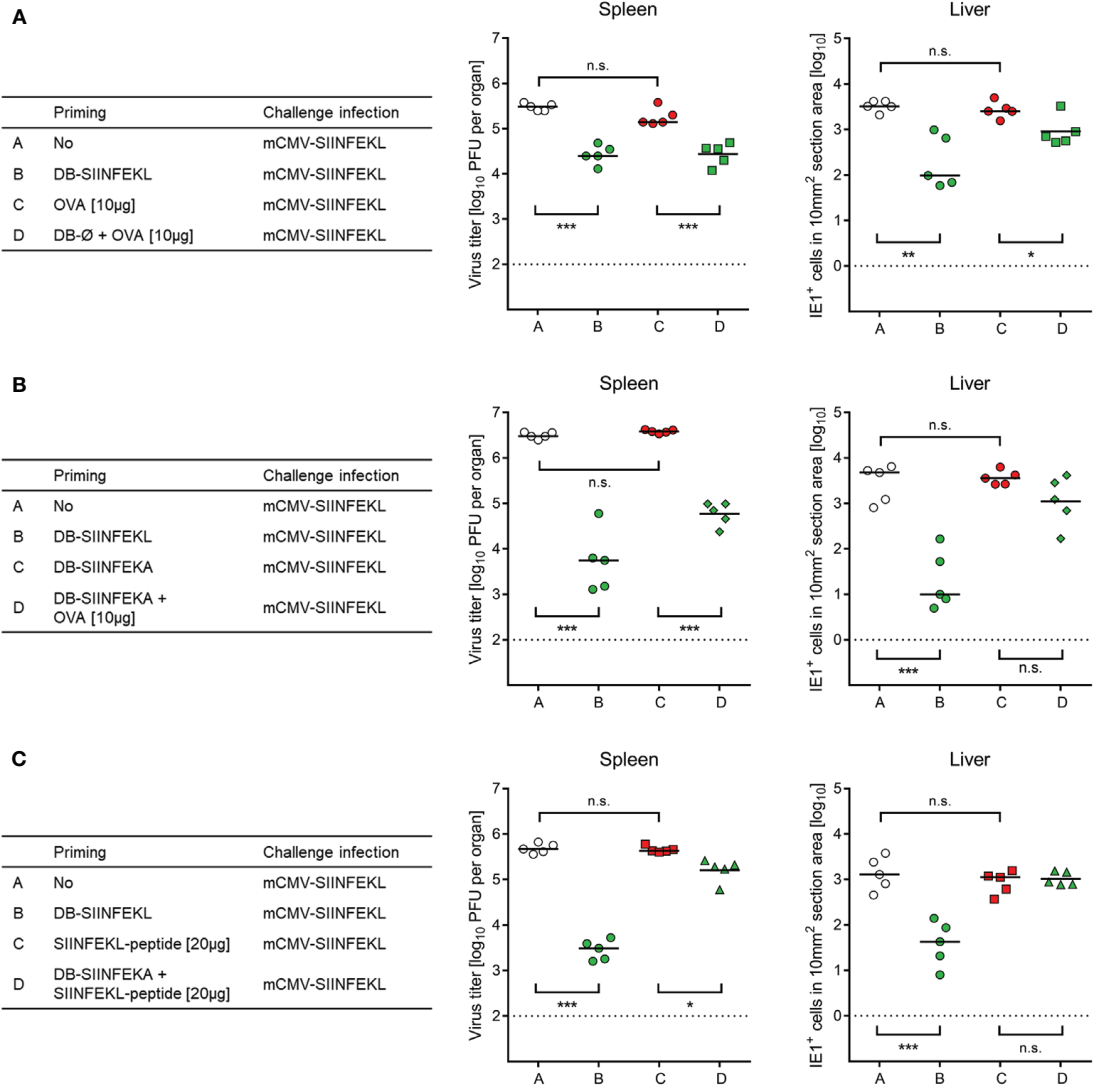
Non-antigenic DBs facilitate priming of a protective response by OVA protein or SIINFEKL peptide. **(A)** Adjuvant role of unmodified DB (DB-Ø) for vaccination with OVA. **(B)** Adjuvant role of non-antigenic DB-SIINFEKA for vaccination with OVA. **(C)** Adjuvant role of non-antigenic DB-SIINFEKA for vaccination with peptide SIINFEKL. Symbols represent data from individually tested mice, with the median values marked. (Open circles), no vaccination. (Green symbols), SIINFEKL-specific priming accomplished. (Red symbols), no SIINFEKL-specific priming accomplished. Significance levels (*) *P* < 0.05, (**) *P* < 0.01, (***) *P* < 0.001, (n.s.), not significant (*P* > 0.05). Dotted lines, detection limits of the assays.

So far, SIINFEKL was included in the DBs as part of a fusion protein with pUL83/pp65, from which it is released after DB uptake through envelope-cell membrane fusion followed by proteasomal processing. One important feature of DBs is their capacity to stimulate maturation and activation of DCs (46), so that they are source of antigen and adjuvant both in one. We therefore wondered if these two roles can be separated. Indeed, whereas purified ovalbumin (OVA) alone failed to prime protective SIINFEKL-specific CD8^+^ T cells, non-antigenic DB-Ø (**Figure 8A**) as well as non-antigenic DB-SIINFEKA (**Figure 8B**) mediated uptake and processing of OVA for priming a protective SIINFEKL-specific response. Finally, non-antigenic DB-SIINFEKA adjuvanted the priming by purified antigenic peptide SIINFEKL (**Figure 8C**). While significance of protection after priming with DB-SIINFEKL was always achieved and in both spleen and liver, DB-adjuvanted priming with OVA protein or SIINFEKL peptide was less efficient in the control of liver infection and did not always reach statistical significance. Thus, providing the antigen as fusion protein within DBs remains the strategy of choice.

In conclusion, this set of experiments has verified efficient priming of protective SIINFEKL-specific CD8^+^ T cells by vaccination with DB-SIINFEKL.

### Vaccination With Recombinant DBs Drives the Epitope-Specific Proliferation of Adoptively Transferred TCR-Transgenic T Cells in Immunocompetent ACT Recipients

**Figure 9:**
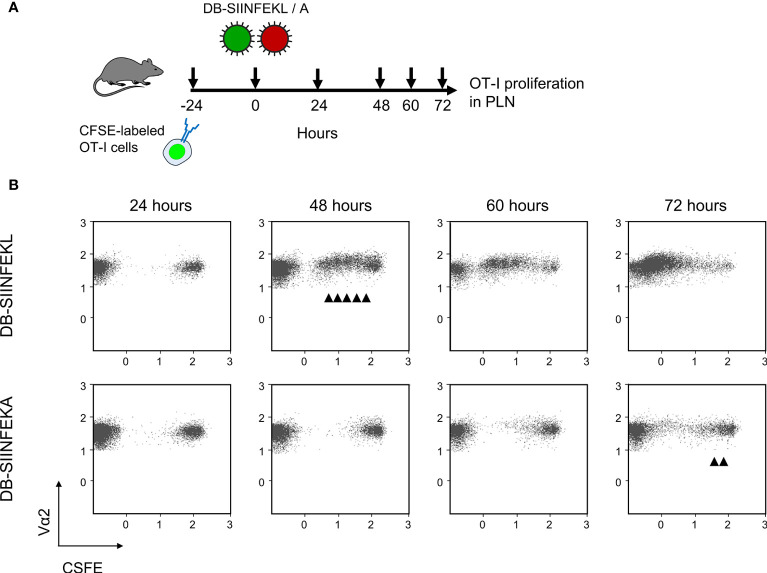
DB-driven epitope-specific proliferation of transferred OT-I cells. **(A)** Experimental protocol. Fluorescence-labeled OT-I cells were transferred intravenously into immunocompetent C57BL/6 mice. Proliferation of OT-I cells recovered from the draining ipsilateral popliteal lymph node (from three mice pooled) was measured at the indicated times after intraplantar priming (vaccination) with 20-μg doses of DB-SIINFEKL or DB-SIINFEKA. **(B)** Cytofluorometric measurement of the loss of the fluorescence label due to the proliferation of OT-I cells over time. (Ordinate) fluorescence specific for the Vα2 TCR chain expressed by OT-I cells. (Abscissa) CFSE fluorescence. For groups of most interest, arrowheads mark cell divisions.

We then combined module-2 and module-3 for testing the capacity of recombinant DBs to drive the proliferation of adoptively transferred OT-I cells in immunocompetent ACT recipients. A pilot experiment showed that sensitization by DB-SIINFEKL was as efficient as infection with mCMV-SIINFEKL in driving the proliferation of OT-I cells in the spleen (**Supplementary Figure S5**). In the main experiment, we looked for the proliferation of transferred CFSE-labeled OT-I cells in the PLN that drains the site of DB vaccine application (**Figure 9A**). Epitope-specific sensitization by DB-SIINFEKL induced more cell divisions and faster than did an epitope-independent sensitization by DB-SIINFEKA (**Figure 9B**). Some delayed activation of OT-I cells by DB-SIINFEKA may result from cytokines expressed in response to antigen-independent DC activation by DBs (43–46).

### TherVac With Recombinant DBs Enhances the Efficacy of Low-Dose Antiviral Immunotherapy in Immunocompromised ACT Recipients

**Figure 10:**
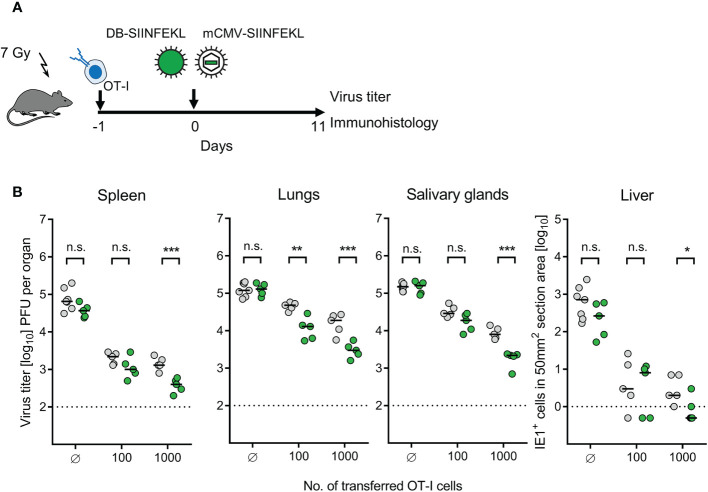
Enhancement of protection by combining ACT with DB-based TherVac. **(A)** Sketch of the protocol. Immunocompromised C57BL/6 mice received either no ACT for control or ACT with 10^2^ or 10^3^ OT-I cells. On the following day, intraplantar TherVac with 20 μg of DB-SIINFEKL and infection with 10^5^ PFU of mCMV-SIINFEKL were performed combined. Control of organ infection was assessed on day 11. **(B)** Virus titers in spleen, lungs, and salivary glands, as well as numbers of infected IE1^+^ liver tissue cells in representative 10-mm^2^ section areas. Symbols (grey-shaded closed circles: no vaccination; green closed circles: vaccination with DB-SIINFEKL) represent data from individually tested mice, with the median values marked. Significance levels (*) *P* < 0.05, (**) *P* < 0.01, (***) *P* < 0.001. (n.s.), not significant (*P* > 0.05). Dotted lines, detection limits of the assays.

The three modules were combined to test if TherVac can enhance the antiviral efficacy of ACT in immunocompromised recipients. TherVac by intraplantar application of DB-SIINFEKL was carried out on the day after intravenous infusion of OT-I cells. A scenario of early virus reactivation was modeled by infection with mCMV-SIINFEKL at the site and time of TherVac (**Figure 10A**). The decisive question was if antiviral T cells in the circulation would migrate to a peripheral site of vaccine application and receive their signals from local antigen presentation.

We expected to see an enhancement of protection by TherVac especially under conditions when low-dose ACT alone is not sufficient. It is our long experience in this model that infection is generally more difficult to control in the lungs compared to spleen and liver [(22) and many publications to follow], and depends on mast cell-derived chemokine CCL5 for enhancing tissue infiltration by protective pulmonary CD8^+^ T cells (70). In accordance with this, a benefit from TherVac after low-dose ACT with just 100 OT-I cells was most significant in the lungs (**Figure 10B**). This is of interest, because the lungs are the clinically most relevant organ site of CMV disease, a fact that reflects the inefficient immune control in the lungs. Thus, enhancing the pulmonary immune response by TherVac may be an option to, at least, reduce the severity of CMV-associated interstitial pneumonia.

### TherVac With Recombinant DBs Enhances the Efficacy of Antiviral Immunotherapy by ACT in HCT Recipients

**Figure 11:**
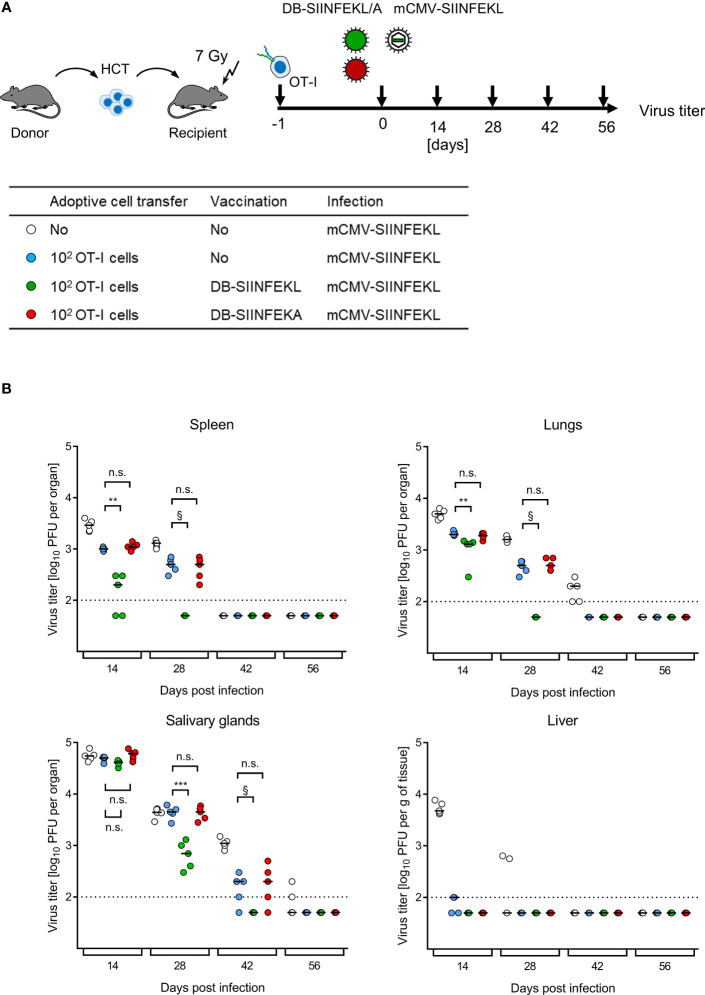
Kinetics of epitope-specific enhancement of protection by combining ACT with DB-based TherVac in HCT recipients. **(A)** Sketch of the protocol and table of experimental groups. Transiently immunocompromised C57BL/6 recipients of syngeneic HCT received ACT with 10^2^ OT-I cells or were left with no ACT for control. On the following day, intraplantar TherVac with 20 μg of DB-SIINFEKL or control TherVac with 20 μg DB-SIINFEKA was combined with infection by 10^5^ PFU of mCMV-SIINFEKL. Control of organ infection was assessed at the indicated times after infection. **(B)** Virus titers in organs indicated. Symbols, color-coded as defined in (A, table), represent data from individually tested mice, with the median values marked. Significance levels (**) *P* < 0.01, (***) *P* < 0.001. (n.s.), not significant (*P* > 0.05). §, de facto significant difference, although calculation of *P* values is pointless when all data of one group in the comparison are below the detection limit of the assay (dotted line).

ACT without HCT has no clinical correlate. The clinical demand is to bridge the critical phase of transient immunodeficiency between hematoablative treatment and endogenous hematopoietic reconstitution by HCT in leukemia/lymphoma patients who are at risk of CMV disease from hCMV reactivation. For studying this situation, we added HCT as module-4 to our mouse model, combining HCT with ACT by OT-I cells, TherVac by recombinant DBs, and mCMV infection. HCT and ACT were performed combined by intravenous transfer of HC and OT-I cells in a mixture. TherVac and infection followed the next day, both by intraplantar application in a mixture (**Figure 11A**).

Endogenous reconstitution of antiviral CD8^+^ T cells by syngeneic HCT eventually led to clearance of productive infection by mCMV-SIINFEKL over time in all organs tested (**Figure 11B**), which is in accordance with previous experience [for reviews, see (7, 8)]. ACT by SIINFEKL-specific OT-I cells reduced virus spread early on and accelerated clearance of productive infection. Specifically, ACT by OT-I cells alone terminated liver infection after HCT by day 14, so that TherVac made no difference. In contrast, in all other organs, TherVac by DB-SIINFEKL, but not by DB-SIINFEKA, further reduced viral replication and accelerated the clearance of productive infection.

**Figure 12:**
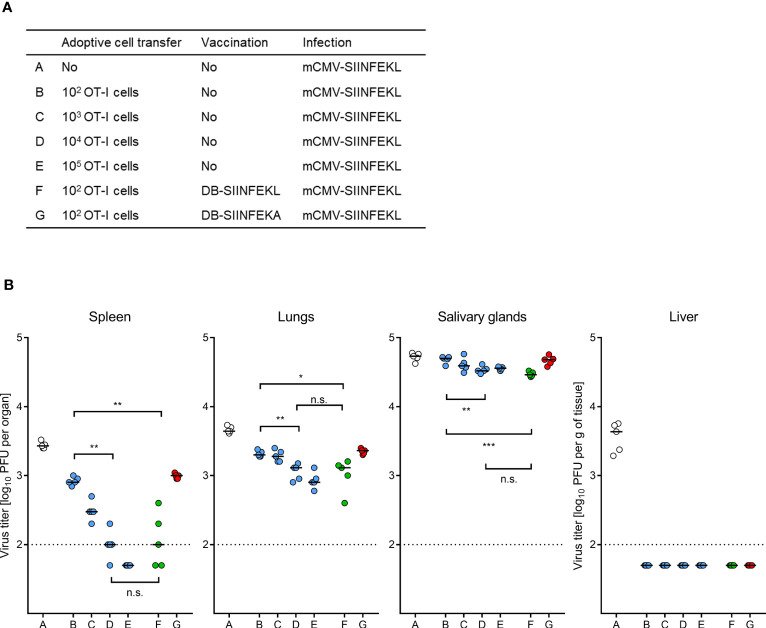
ACT dose-benefit from TherVac in HCT recipients. **(A)** Table of experimental groups A–G. For the protocol, see the sketch in Figure 11A, with the modification that ACTs alone were performed with graded numbers of OT-I cells. Organ infection was assessed on day 14 after infection with mCMV-SIINFEKL. **(B)** Virus titers in organs indicated. Symbols (open circles: no ACT, blue closed circles: ACT alone, green closed circles: ACT and TherVac with DB-SIINFEKL, red closed circles: ACT and control TherVac with DB-SIINFEKA) represent data from individually tested mice, with the median values marked. Significance levels (*) *P* < 0.05, (**) *P* < 0.01, (***) *P* < 0.001. (n.s.), not significant (*P* > 0.05). Dotted lines, detection limit of the assay.

We finally performed ACT in HCT recipients with graded numbers of OT-I cells to determine the benefit from TherVac by DB-SIINFEKL in terms of ACT cell numbers required for equivalent protection (**Figure 12A**). Control of mCMV-SIINFEKL infection by 100 OT-I cells combined with TherVac by DB-SIINFEKL, but not by DB-SIINFEKA, was equivalent in all tested organs to infection control by 10,000 OT-I cells in absence of TherVac (**Figure 12B**). Thus, in this experiment, TherVac gave a 100-fold benefit in terms of ACT cell numbers saved.

## Discussion

This study in the mouse model has provided proof-of-concept for enhancing the efficacy of low-dose ACT against CMV disease in an HCT setting by TherVac: no more, no less. This may encourage clinical investigation and trials, just as the first demonstration of CMV-specific ACT in the mouse model (22–24) has intellectually paved the way to clinical ACT as an immune-therapeutical approach to the prevention of disease from hCMV reactivation in HCT patients [for a review on medical translation of results from animal CMV models, see (9)].

In the weakness of any reductionistic approach in animal models to never be able to reproduce the clinical reality in all its complexity (71) lies also the strength of a less obstructed view on fundamental principles. The outcome of CMV infection in HCT patients is highly individual, as many variables, which are difficult to control, determine the individual fate. To begin with, an underestimated and sometimes ignored factor is a pre-existing defect from the primary disease, that is, a hematopoietic malignancy and associated chemotherapy. Leukemia relapse after therapy by HCT is a major concern. Thus, in histocompatibility antigen-mismatched “allogeneic” HCT, mature T cells are not depleted from the transplant to maintain a graft-*versus*-leukemia (GvL) response, taking the risk of a GvH response, which is associated with a higher risk of CMV reactivation (72). In addition, the CMV status of donor and recipient decides on whether virus reactivation occurs within the transplanted HC or within the recipient’s own tissues, or both [reviewed in (2)], and the individual’s genetic constitution and infection history defines the latent CMV genome load and incidence of reactivation [discussed in (73)]. The time of virus reactivation in an individual HCT recipient is not predictable, and viral loads after reactivation vary dramatically between individuals (10). To conclude this certainly not comprehensive list of variables, genetic and phenotypic differences between hCMV strains/variants, which are rarely typed in HCT clinical routine, can have a fundamental impact on cell-type tropism and thus on the pathogenicity of the reactivating virus (74, 75). With all this in mind, it becomes evident that no experimental animal model will ever perfectly suit human CMV disease in any individual HCT recipient.

Pre-emptive immunotherapy of hCMV reactivation by ACT with virus-specific CD8^+^ T cells is an option to prevent CMV organ disease in HCT recipients who are latently infected (constellation D^-^R^+^), who receive an HC transplant derived from a latently infected donor (constellation D^+^R^-^), or who combine both risk factors (constellation D^+^R^+^). In HCT, the risk of virus reactivation is highest in D^-^R^+^ patients who do not receive donor immunity with the transplant, as well as in D^+^R^+^ patients who receive a T cell-depleted transplant for avoiding GvH disease [for a review, see (2)]. ACT with purified virus-specific CD8^+^ T-cell preparations avoids GvH disease while selectively targeting infected cells. It is a general alternative to pharmacotherapy with antiviral drugs for avoiding myelosuppressive or nephrotoxic side effects, and it is the last resort to fight infection by virus strains/variants that are refractory to antiviral medication (15–17, 21) from the outset or that develop resistance and become selected under longer-term treatment (see also the Introduction).

In case of the HCT constellations with D^+^ (see above), the HCT donor is the first choice as a source of virus-specific CD8^+^ T cells for ACT. HLA/MHC class-I restriction of CD8^+^ T-cell function does here not seriously pose a limitation, because an unrelated HCT donor and the recipient need to be HLA type-matched to share HLA antigens as complete as possible for avoiding GvH disease. An expanding list of viral peptides identified to be presented by more common HLA class-I molecules allows purification of viral epitope-specific donor CD8^+^ T cells for ACT by various cell sorting techniques [(32–35), for reviews, see (76, 77)]. In case of HCT constellations with an hCMV-negative donor (D^-^), CD8^+^ T cells can be derived from an unrelated, hCMV-experienced third-party donor sharing HLA molecules with the HCT/ACT recipient (77). As a more recent strategy of ACT, naïve or memory CD8^+^ T cells can be equipped with an engineered, transgenic TCR specific for an HLA class-I presented antigenic peptide, as studied in viral and tumor models [(38–40), for reviews, see (78, 79)]. The first clinical approaches of ACT for preventing hCMV infection in HCT recipients were performed with cell culture-propagated, and thereby highly expanded, clonal CD8^+^ CTLL (18, 19). By directly comparing ACTs with CTLL and *ex vivo* isolated CD8^+^ T cells of the same specificity, studies in the mouse model revealed that the benefit from expanding antiviral cells to high cell numbers by recombinant IL2 is largely dashed by a loss of per-cell antiviral activity (30, 31). More recent studies indicated that autocrine IL2 induced by cultivation in the presence of IL7 and IL15, but also co-stimulatory signals, can improve persistence and proliferation potential of cell culture-propagated CD8^+^ T cells upon ACT [(80), for a review, see (81)].

It is an underappreciated finding that combining protective epitope specificities failed to improve the antiviral efficacy of ACT (82). This can be explained by the fact that an infected cell cannot “die two deaths”. This insight is of practical importance, as sorting of CD8^+^ T cells specific for a single type of antigenic peptide presented in both the ACT donor and recipient, or a single type of engineered viral epitope-specific TCR, should suffice for the control of the infection upon ACT. It is also worth to note that protective efficacy upon ACT is not linked to the immunodominance of an antigenic peptide in the natural immune response against the virus (58, 83). It rather depends on the structural avidity of the TCR and the functional avidity with which CD8^+^ T cells interact with pMHC-I complexes on the surface of infected cells (78, 84, 85). This parameter becomes most important when viral immune evasion proteins limit antigen presentation. Specifically, a functional avidity corresponding to the recognition of target cells exogenously loaded with ≤10^-9^ M of an antigenic peptide is required for the recognition of mCMV-infected cells when immune evasion molecules are expressed (55).

A clinical application of ACT in HCT recipients diagnosed to have latent hCMV reactivated has so far not become a routine therapy, mainly because the logistics for providing sufficiently high donor cell numbers is demanding. Already shortly after the first experimental ACT with CD8^+^ T cells in the mouse model of CMV infection of the immunocompromised host (22), we pursued the idea to expand low numbers of transferred cells within the mCMV-infected recipients. Specifically, inspired by earlier work on IL2-enhanced ACT for cancer therapy (86–88), we showed that recombinant IL2 administered in 12-hour intervals resulted in enhanced antiviral protection equivalent to daily doublings of the effector cell population (23). However, besides enormous costs for recombinant cytokines, meanwhile including also IL7, -12, -15, and -21, adverse side effects by unspecific activation need to be considered (81).

Here we have pursued the alternative strategy of expanding adoptively transferred cells in CMV-infected, combined HCT and ACT recipients by TherVac, a concept discussed also for tumor therapy [reviewed in (81)]. As a model for CD8^+^ T cells with transgenic TCR, modeling ACT with cells from CMV-negative donors, we used OT-I cells specific for the pMHC-I complex SIINFEKL-K^b^. Syngeneic HCT was performed with C57BL/6 mice (*H-2^b^* haplotype) as donors and recipients in order not to complicate the model by consequences of mismatch in major or minor histocompatibility antigens. According to most recent findings, such mismatches, rather than causing a GvH reaction, inhibit the reconstitution of protective high-avidity CD8^+^ T cells by inducing non-cognate transplantation tolerance (67, 68). Our data show an exquisite epitope-specific function of OT-I cells. ACT controlled virus mCMV-SIINFEKL, but not the epitope-loss mutant mCMV-SIINFEKA, in the tissues of immunocompromised recipients.

For TherVac, we chose hCMV DBs, which represent non-infectious, DNA-free subviral particles consisting of enveloped viral tegument proteins (43, 69). DBs can be modified to package recombinant tegument protein pp65/UL83 containing integrated immunogenic peptides of interest (45). As an advantage of the DB concept of vaccination, envelope glycoprotein complexes mediate the fusion of DBs with the cell membrane of target cells and deliver the tegument proteins directly into the cytosol for antigen presentation in the MHC-I pathway (43–45). Thus, also cells of non-hematopoietic cell lineages can present antigenic peptides to already primed CD8^+^ T cells used for ACT. In addition, the DB entry process directly activates professional antigen-presenting cells (profAPCs), such as DC (46) for the priming of naïve CD8^+^ T cells, so that a DB-based vaccine does not require adjuvantation (53). For use as a vaccine against hCMV, new generations of DBs (89, 90) have the additional advantage of priming an antibody response directed against the virion trimeric and pentameric entry complexes in order to cover hCMV strains that differ in cell tropism. Our data show an exquisite epitope-specific expansion of OT-I cells in that they proliferated and exerted an enhanced antiviral protection only after TherVac with DB-SIINFEKL but not with DB-SIINFEKA. Although we have here chosen recombinant DBs for TherVac, it is important to emphasize that the concept of TherVac is, of course, not limited to the use of a DB-based vaccine, but is open for alternative vaccination strategies.

A difference between vaccination of immunocompetent hosts and TherVac in immunocompromised recipients of combined HCT and ACT is the transient shortness of hematopoietic lineage-derived profAPCs at early times after HCT. As we have shown previously in a sex-chimeric mouse model of HCT with male *sry* ^+^ donors and female *sry^-^* recipients, CD11c^+^ DC are preferentially lost in the recipients after hematoablative treatment and replaced with donor-derived cells only with delay (91, 92). So, immediate expansion of transferred antiviral CD8^+^ T cells depends on direct antigen expression by non-hematopoietic cells targeted by the vaccine. It was therefore an open question if limited numbers of transferred cells would at all encounter host cells presenting the vaccine antigen for driving clonal expansion. Our data show that TherVac amplifies the protective efficacy of limited numbers of OT-I cells even when ACT is performed intravenously and TherVac locally into a footpad. As we have discussed recently (55), intraplantar vaccine application in the mouse is a good model for subcutaneous or intramuscular vaccine application into the upper arm of vaccinees, the favored site for routine vaccination of humans. This finding increases the chances for a clinical translation to TherVac in human recipients of HCT-ACT.

Let us speculate on a translation of our findings to a clinical application. Clinical studies discussed already above (32–36) have shown that low-dose ACT works, in principle, with no need for TherVac. This might be raised as an argument against TherVac. However, a further enhancement by TherVac may be beneficial to control infection also at “immune-privileged” sites at which infection is more difficult to control. Notably, these studies have also revealed that control of infection depends on massive clonal expansion of the initially few cells transferred, and this expansion depends on stimulation by cognate antigen. As we have shown here, OT-I cells expand in the infected recipients within 10 days from 100 cells to more than 10^6^ cells (recall **Figure 4B**) when driven by the cognate epitope SIINFEKL. The dependence on antigen implies that the expansion, and thus the efficacy of ACT, is low when antigen presentation is limited, for instance, at a very early stage of virus reactivation, which is a stochastic event occurring in only few cells (93, 94). Likewise, an expansion will predictably be limited, when viral replication, and hence antigen presentation, is inhibited by antiviral drugs, so that combining antiviral pharmacotherapy with immunotherapy by ACT makes little sense. In such scenarios, specifically in a phase when antiviral medication is planned to taper off, TherVac-enhanced ACT may be an option to prevent a relapse of infection.

Altogether, the possibility to apply the vaccine locally and without adjuvant, combined with strict epitope-specificity of the enhancement of the protective effector function, makes TherVac superior to cytokine cocktails for the post-ACT clonal expansion of protective antiviral CD8^+^ T cells and gives new options for the prevention of CMV disease in HCT recipients.

## Biosafety Statement

The work was done according to German federal law GenTG and BioStoffV. The generation of recombinant mCMV and hCMV was approved by the ‘Struktur- und Genehmigungsdirektion Süd’ (SDG, Neustadt, Germany), permission numbers 24.1-886.3 and 25.2-886.3, respectively.

## Data Availability Statement

The original contributions presented in the study are included in the article/**Supplementary Material**. Further inquiries can be directed to the corresponding author.

## Ethics Statement

The animal study was reviewed and approved by the ethics committee of the “Landesuntersuchungsamt Rheinland-Pfalz” according to German federal law §8 Abs. 1 TierSchG (animal protection law), permission numbers 177-07/G 10-1-52.

## Author Contributions

MR and NL are responsible for conception and design of the study, analysis, and interpretation of the data. RH and BP provided essential material and designed parts of the study. KG, JP, SB, KF, SK, NB, and NL conducted the work and analyzed the data. MR wrote the first draft of the manuscript. NL wrote sections of the manuscript. All authors contributed to manuscript revision and read and approved the submitted version.

## Funding

This work was supported by the Deutsche Forschungsgemeinschaft (DFG), Clinical Research Group KFO 183, individual project 8 (KG, BP, MR, and NL) and SFB1292, individual projects TP11 (SB, MR, and NL) and TP14 (RH), and by the Wilhelm Sander Stiftung application number 2020.003.1 (BP).

## Conflict of Interest

The authors declare that the research was conducted in the absence of any commercial or financial relationships that could be construed as a potential conflict of interest.

## Publisher’s Note

All claims expressed in this article are solely those of the authors and do not necessarily represent those of their affiliated organizations, or those of the publisher, the editors and the reviewers. Any product that may be evaluated in this article, or claim that may be made by its manufacturer, is not guaranteed or endorsed by the publisher.

## Supplementary Material

Supplementary Figure 1 Viral replicative fitness. Virus growth *in vivo* in absence of immune control was determined in the indicated organs of C57BL/6 mice that were immunocompromised by sublethal total-body γ-irradiation with a single dose of 7 Gy, followed by intraplantar infection with 1 × 10^5^ PFU of the indicated viruses. At the indicated times after infection, virus growth parameters were measured, specifically burden of infectious virus (PFU in spleen, lungs, and salivary glands) or numbers of infected cells in representative 10-mm^2^ areas of liver tissue sections. Symbols represent data from three to four mice tested individually per time of assay. Graphs show log-linear regression lines (based on data from all time points) and their corresponding 95% confidence regions bordered by dotted lines. Viral doubling times (*vDT*) were calculated based on the slopes of the regression lines according to the formula *vDT* = log2/*a*. The 95% confidence intervals of *vDT* are given in parentheses.

Supplementary Figure 2 Phenotypic and functional characterization of TCR-transgenic OT-I cells. **(A)** Cytofluorometric quantitation of cells expressing SIINFEKL-specific Vα2Vβ5 TCR among CD8^+^ T cells derived from the spleen of OT-I mice. **(B)** Determination of the functional avidity of OT-I cells in an IFNγ-ELISpot assay with EL-4 stimulator cells exogenously loaded with synthetic peptide SIINFEKL in the graded molar concentrations indicated. Bars represent numbers of OT-I cells responding with IFNγ secretion. Error bars represent 95% confidence intervals. (Top panel), cumulative avidity distribution revealing frequencies of OT-I cells responding to the indicated peptide concentration tested, which includes cells that would also respond to lower concentrations. (Bottom panel), Gaussian-like avidity distribution revealing frequencies of OT-I cells responding exactly to the peptide concentration indicated. This distribution is deduced from the cumulative avidity distribution values by plotting the difference to the respective lower-concentration frequency.

Supplementary Figure 3 Lymphoid homing and OT-I proliferation driven by local infection. Proliferation of intravenously transferred, CFSE-labeled OT-I cells (transfer on day -1) in popliteal lymph nodes (PLN) of C57BL/6 mice, determined at 60 h after unilateral intraplantar infection with mCMV-SIINFEKL expressing the cognate epitope. (Top panel), lack of OT-I proliferation in the contralateral PLN not draining the site of infection. (Bottom panel), OT-I proliferation in the ipsilateral PLN draining the site of infection. FSC, forward scatter. CFSE, fluorescence intensity. Arrowheads mark cell divisions.

Supplementary Figure 4 OT-I cells fail to control liver infection when the cognate epitope is not expressed. Corresponding to **Figure 4A**, 2C-IHC images of liver tissue sections show extensive virus spread and random distribution of liver-infiltrating OT-I cells after infection of immunocompromised C57BL/6 mice with mCMV-SIINFEKA not expressing the cognate epitope. (Left images), low magnification overviews. (Right images), resolved to greater detail by higher magnification. (Black staining), CD8^+^ T cells. (Red staining), infected liver cells. Bar markers, 50 μm. For additional information, see the legend of **Figure 4**.

Supplementary Figure 5 Equivalence of OT-I proliferation driven by mCMV-SIINFEKL and DB-SIINFEKL. Proliferation of intravenously transferred, CFSE-labeled OT-I cells (transfer on day -1) in the spleen of C57BL/6 mice, determined at 72 h after infection or vaccination. FSC, forward scatter. CFSE, fluorescence intensity. Arrowheads mark cell divisions.
