# Supplementary figures and images for: Therapeutic Vaccination of Hematopoietic Cell Transplantation Recipients Improves Protective CD8 T-Cell Immunotherapy of Cytomegalovirus Infection
Source: Front Immunol. 2021 Aug 19;12:694588. doi: 10.3389/fimmu.2021.694588 (PMC8416627; doi:10.3389/fimmu.2021.694588)

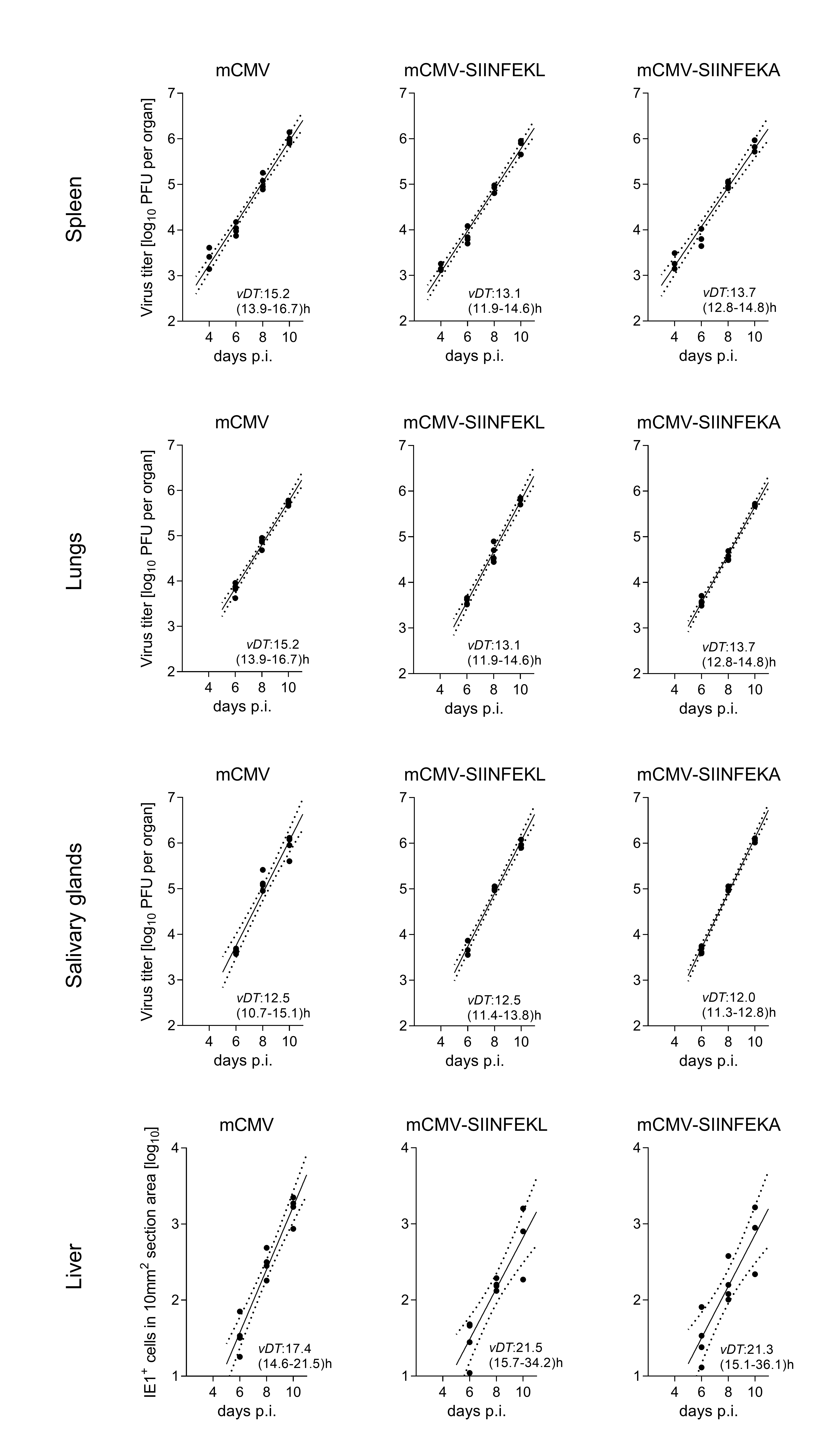

Supplement: Supplementary Figure 1 — Viral replicative fitness. Virus growth in vivo in absence of immune control was determined in the indicated organs of C57BL/6 mice that were immunocompromised by sublethal total-body γ-irradiation with a single dose of 7 Gy, followed by intraplantar infection with 1 × 10^5^ PFU of the indicated viruses. At the indicated times after infection, virus growth parameters were measured, specifically burden of infectious virus (PFU in spleen, lungs, and salivary glands) or numbers of infected cells in representative 10-mm2 areas of liver tissue sections. Symbols represent data from three to four mice tested individually per time of assay. Graphs show log-linear regression lines (based on data from all time points) and their corresponding 95% confidence regions bordered by dotted lines. Viral doubling times (vDT) were calculated based on the slopes of the regression lines according to the formula vDT = log2/a. The 95% confidence intervals of vDT are given in parentheses. [file Image_1.tif]

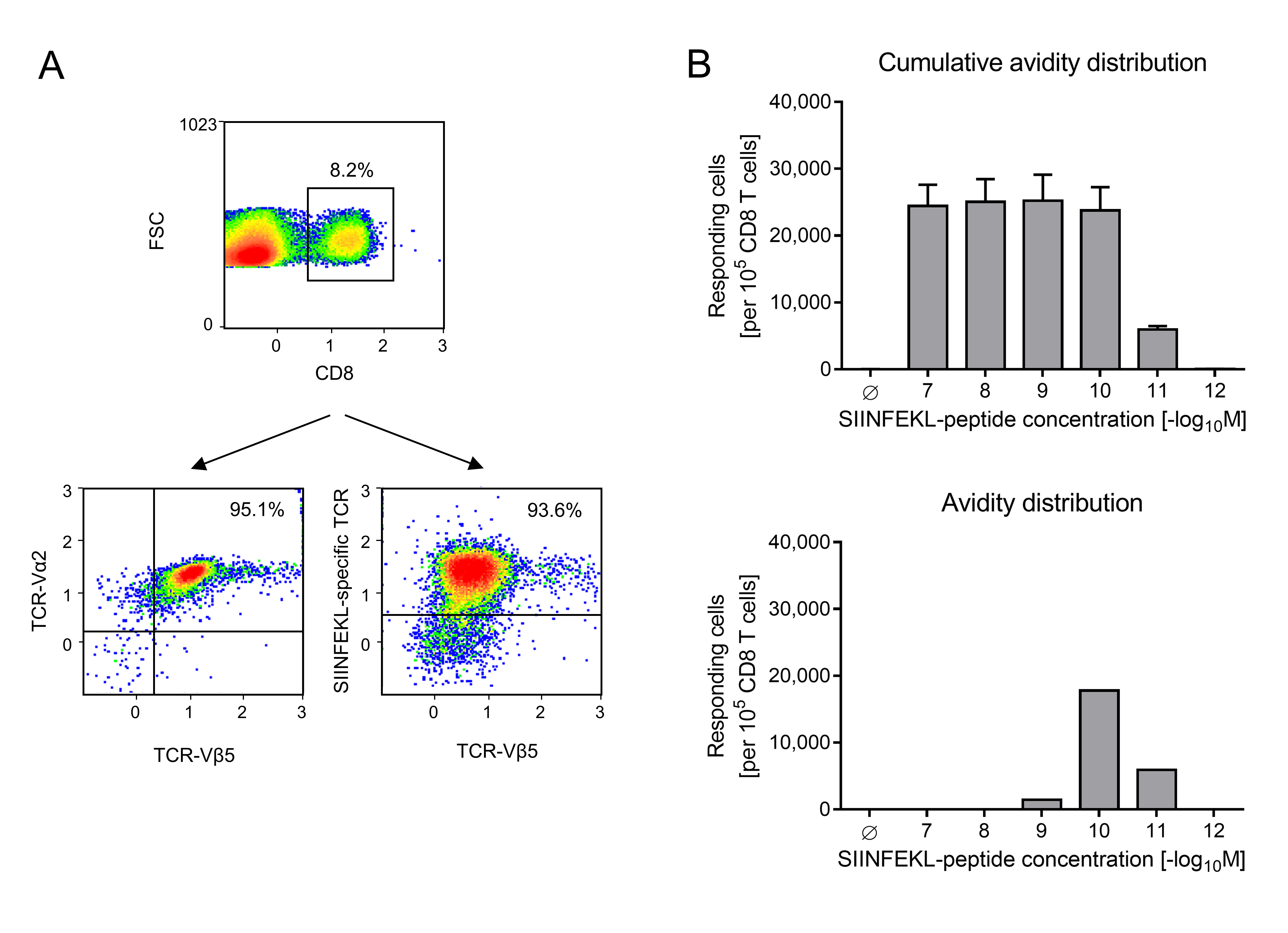

Supplement: Supplementary Figure 2 — Phenotypic and functional characterization of TCR-transgenic OT-I cells. (A) Cytofluorometric quantitation of cells expressing SIINFEKL-specific Vα2Vβ5 TCR among CD8+ T cells derived from the spleen of OT-I mice. (B) Determination of the functional avidity of OT-I cells in an IFNγ-ELISpot assay with EL-4 stimulator cells exogenously loaded with synthetic peptide SIINFEKL in the graded molar concentrations indicated. Bars represent numbers of OT-I cells responding with IFNγ secretion. Error bars represent 95% confidence intervals. (Top panel), cumulative avidity distribution revealing frequencies of OT-I cells responding to the indicated peptide concentration tested, which includes cells that would also respond to lower concentrations. (Bottom panel), Gaussian-like avidity distribution revealing frequencies of OT-I cells responding exactly to the peptide concentration indicated. This distribution is deduced from the cumulative avidity distribution values by plotting the difference to the respective lower-concentration frequency. [file Image_2.tif]

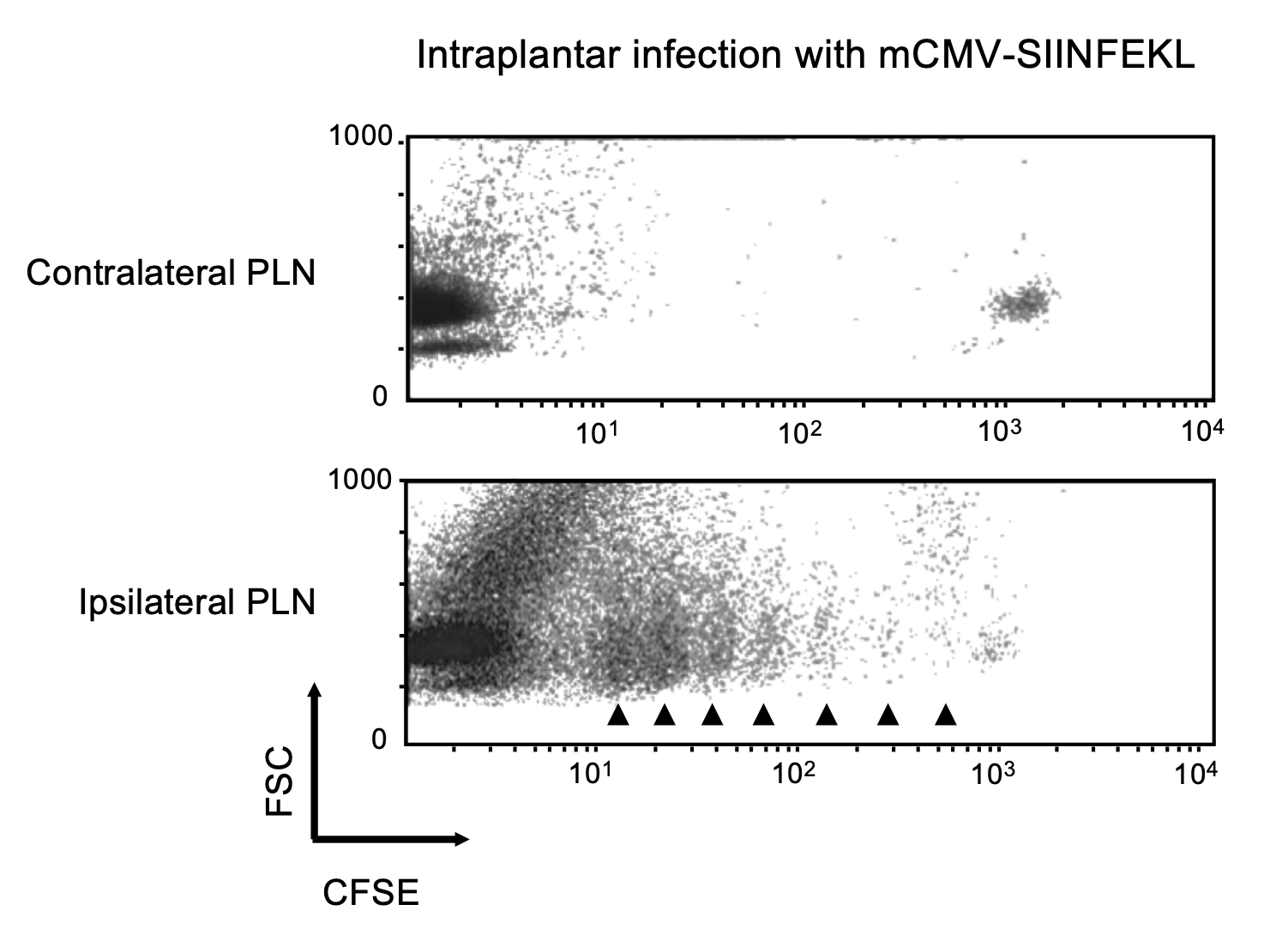

Supplement: Supplementary Figure 3 — Lymphoid homing and OT-I proliferation driven by local infection. Proliferation of intravenously transferred, CFSE-labeled OT-I cells (transfer on day -1) in popliteal lymph nodes (PLN) of C57BL/6 mice, determined at 60 h after unilateral intraplantar infection with mCMV-SIINFEKL expressing the cognate epitope. (Top panel), lack of OT-I proliferation in the contralateral PLN not draining the site of infection. (Bottom panel), OT-I proliferation in the ipsilateral PLN draining the site of infection. FSC, forward scatter. CFSE, fluorescence intensity. Arrowheads mark cell divisions. [file Image_3.tiff]

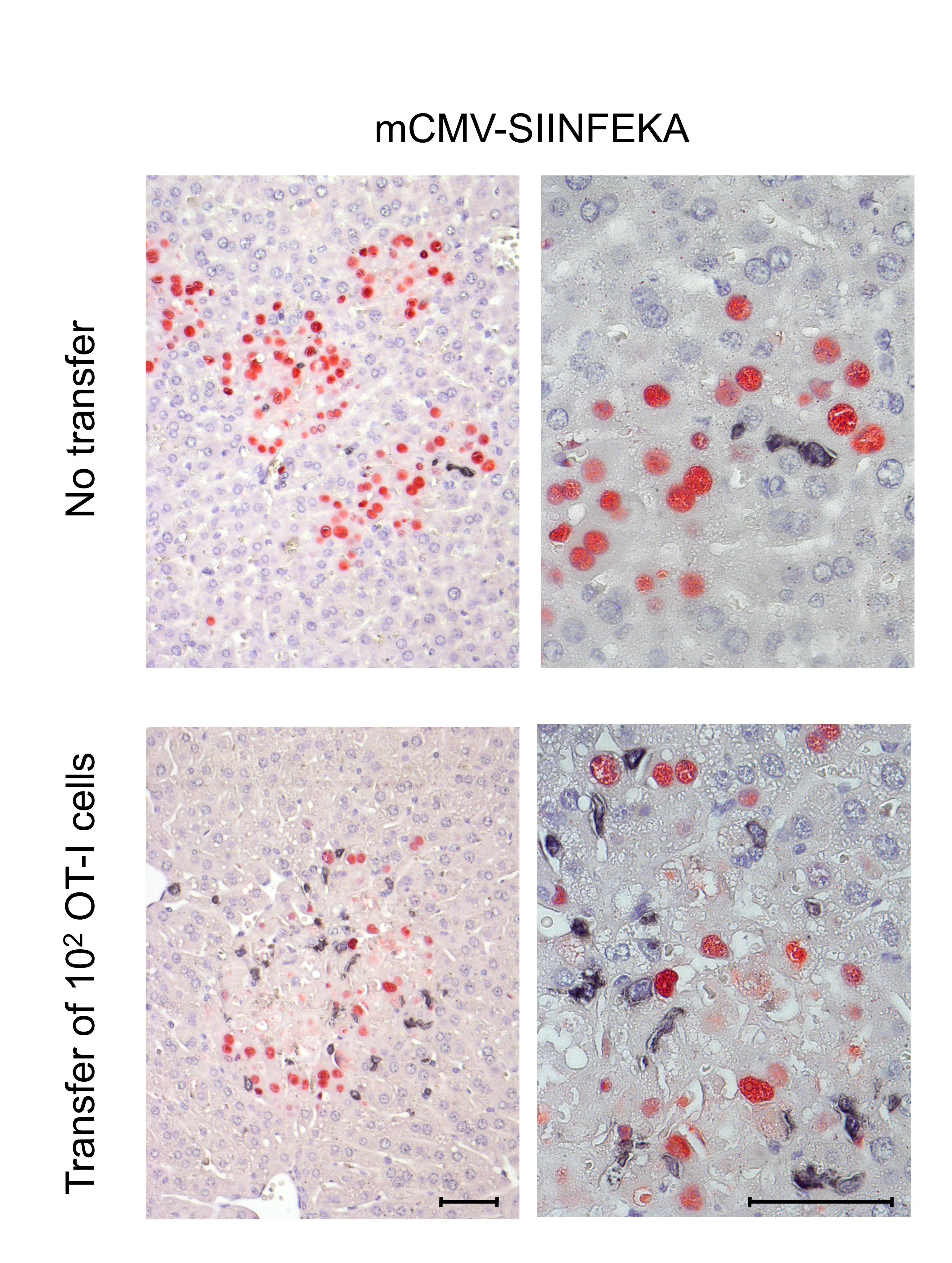

Supplement: Supplementary Figure 4 — OT-I cells fail to control liver infection when the cognate epitope is not expressed. Corresponding to Figure 4A, 2C-IHC images of liver tissue sections show extensive virus spread and random distribution of liver-infiltrating OT-I cells after infection of immunocompromised C57BL/6 mice with mCMV-SIINFEKA not expressing the cognate epitope. (Left images), low magnification overviews. (Right images), resolved to greater detail by higher magnification. (Black staining), CD8+ T cells. (Red staining), infected liver cells. Bar markers, 50 μm. For additional information, see the legend of Figure 4. [file Image_4.tif]

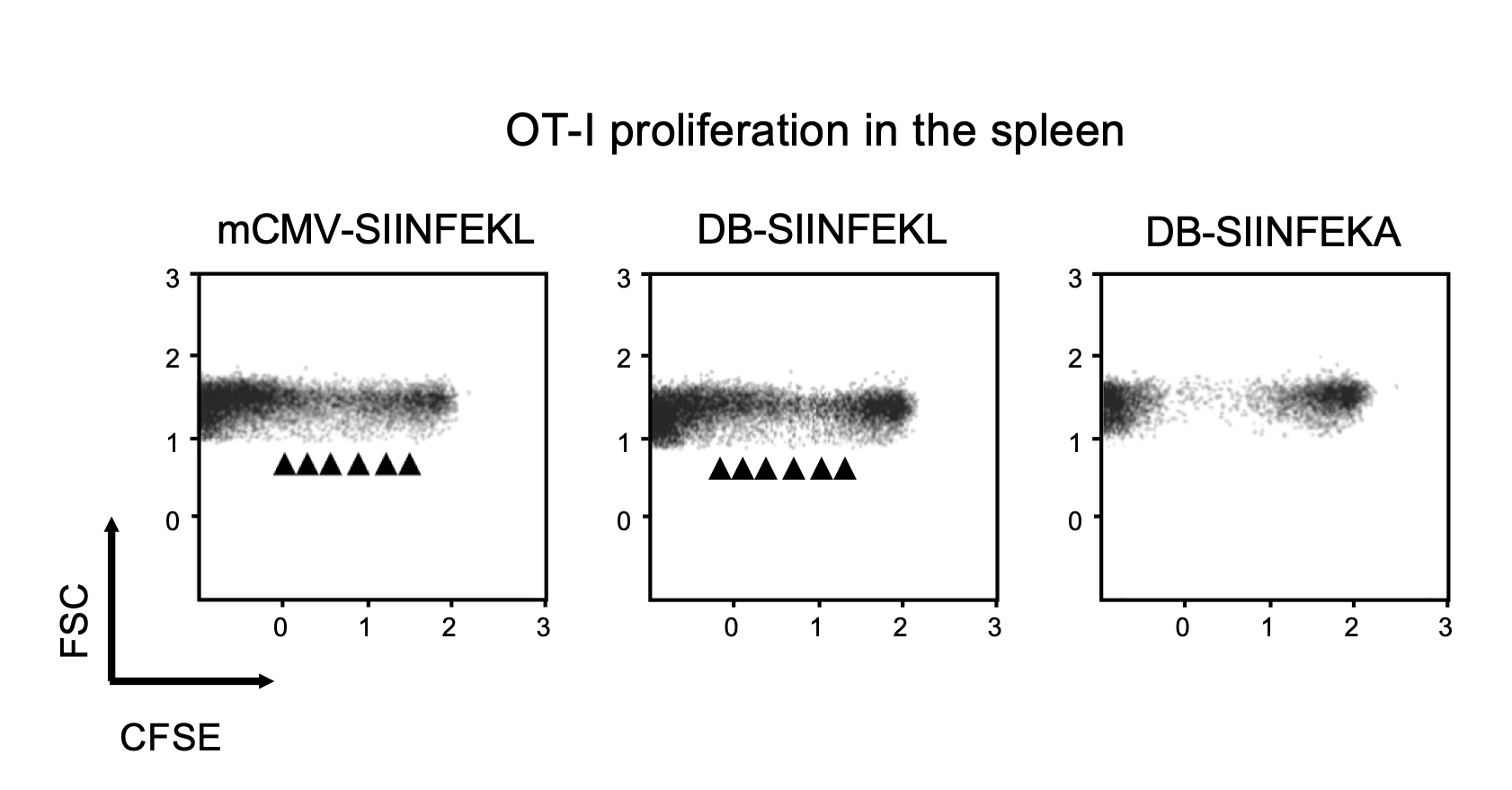

Supplement: Supplementary Figure 5 — Equivalence of OT-I proliferation driven by mCMV-SIINFEKL and DB-SIINFEKL. Proliferation of intravenously transferred, CFSE-labeled OT-I cells (transfer on day -1) in the spleen of C57BL/6 mice, determined at 72 h after infection or vaccination. FSC, forward scatter. CFSE, fluorescence intensity. Arrowheads mark cell divisions. [file Image_5.tiff]
